# The Gut Microbiome of an Indigenous Agropastoralist Population in a Remote Area of Colombia with High Rates of Gastrointestinal Infections and Dysbiosis

**DOI:** 10.3390/microorganisms11030625

**Published:** 2023-02-28

**Authors:** Simone Kann, Kirsten Eberhardt, Rebecca Hinz, Norbert Georg Schwarz, Juan Carlos Dib, Andres Aristizabal, Gustavo Andrés Concha Mendoza, Ralf Matthias Hagen, Hagen Frickmann, Israel Barrantes, Bernd Kreikemeyer

**Affiliations:** 1 Department for Research and Development, Bernhard Nocht Institute for Tropical Medicine, 20359 Hamburg, Germany; 2 Department of Tropical Medicine, Bernhard Nocht Institute for Tropical Medicine, 20359 Hamburg, Germany; 3 I. Department of Medicine, University Medical Center Hamburg-Eppendorf, 20251 Hamburg, Germany; 4 Division of Hygiene and Infectious Diseases, Institute of Hygiene and Environment, 20539 Hamburg, Germany; 5 SYNLAB Medizinisches Versorgungszentrum Hamburg GmbH, 22083 Hamburg, Germany; 6 Independent Researcher, 67459 Böhl-Iggelheim, Germany; 7 Department of Medicine, Fundación Universidad de Norte, Baranquilla 080001, Colombia; 8 Tropical Health Foundation, Santa Marta 470003, Colombia; 9 Institución Prestadora de Servicios de Salud Indígena, Dusakawi IPSI, Valledupar 200001, Colombia; 10 Department of Microbiology and Hospital Hygiene, Bundeswehr Central Hospital Koblenz, 56070 Koblenz, Germany; 11 Department of Microbiology and Hospital Hygiene, Bundeswehr Hospital Hamburg, 20359 Hamburg, Germany; 12 Institute for Medical Microbiology, Virology and Hygiene, University Medicine Rostock, 18057 Rostock, Germany; 13 Research Group Translational Bioinformatics, Institute for Biostatistics and Informatics in Medicine und Aging Research, University Medicine Rostock, 18057 Rostock, Germany

**Keywords:** next generation sequencing, gut microbiome, remote tropical area, Indigenous, Colombia, dysbiosis, gastroenteritis

## Abstract

An Indigenous agropastoralist population called the Wiwa from the Sierra Nevada de Santa Marta, in North-East Colombia, shows high rates of gastrointestinal infections. Chronic gut inflammatory processes and dysbiosis could be a reason, suggesting an influence or predisposing potential of the gut microbiome composition. The latter was analyzed by 16S rRNA gene amplicon next generation sequencing from stool samples. Results of the Wiwa population microbiomes were associated with available epidemiological and morphometric data and compared to control samples from a local urban population. Indeed, locational-, age-, and gender-specific differences in the Firmicutes/Bacteriodetes ratio, core microbiome, and overall genera-level microbiome composition were shown. Alpha- and ß-diversity separated the urban site from the Indigenous locations. Urban microbiomes were dominated by *Bacteriodetes*, whereas Indigenous samples revealed a four times higher abundance of *Proteobacteria*. Even differences among the two Indigenous villages were noted. PICRUSt analysis identified several enriched location-specific bacterial pathways. Moreover, on a general comparative scale and with a high predictive accuracy, we found *Sutterella* associated with the abundance of enterohemorrhagic *Escherichia coli* (EHEC), *Faecalibacteria* associated with enteropathogenic *Escherichia coli* (EPEC) and helminth species *Hymenolepsis nana* and *Enterobius vermicularis*. *Parabacteroides*, *Prevotella*, and *Butyrivibrio* are enriched in cases of salmonellosis, EPEC, and helminth infections. Presence of *Dialister* was associated with gastrointestinal symptoms, whereas *Clostridia* were exclusively found in children under the age of 5 years. *Odoribacter* and *Parabacteroides* were exclusively identified in the microbiomes of the urban population of Valledupar. In summary, dysbiotic alterations in the gut microbiome in the Indigenous population with frequent episodes of self-reported gastrointestinal infections were confirmed with epidemiological and pathogen-specific associations. Our data provide strong hints of microbiome alterations associated with the clinical conditions of the Indigenous population.

## 1. Introduction

The gut microbiome is a diverse community of microbial eukaryotes, viruses, archaea, and, mostly, bacteria, and plays a fundamental role in human biology [1,2,3]. Its composition is associated with human physiology and human developmental processes, and has an important role in human health and disease [4]. There are numerous studies providing evidence of gut microbial influence on immunity, metabolism, neurodegenerative processes, and even human psychology [5,6,7,8]. Therefore, it is of scientific interest to investigate the microbiota of humans living in different continents and countries all over the world. There is increasing evidence of an existing “core gut microbiome” which is conserved and pre-dates the split of Neanderthals and modern humans [9]. However, with an increasing displacement of paleolithic lifestyles and subsistence strategies, there is finally a widely appreciated risk of losing ancient microorganisms, such as fiber-degrading bacteria, due to fast-paced globalization and industrialization [10].

Thus, numerous studies have investigated and shown differences in the gut microbiota composition, reflecting different environmental conditions, geographic region and ethnicity, dietary habits, and general lifestyle [11]. Moreover, subsistence, split into modern urban, pastoralist, agropastoralist, and hunter–gatherer types, had a strong influence on the gut microbiome composition [10]. Several studies have investigated such gut microbiome associations and drifts in an Inuit population, populations from Cameroon, Tanzania, Botswana, Burkina Faso, Nigerian Fulani, Mexican Me’phaa, Central African Republic BaAka, and Bantu, and Yanomami from different locations of the Americas [12,13,14,15,16,17,18,19,20].

Comparative microbiome composition information from Indigenous tribes in Colombia is still lacking. Recently, a cross-sectional study including healthy individuals of both genders representing the general population of Medellin in Colombia was conducted and results were compared to Europeans, Americans, and Asians [21]. However, Colombia is known for its biodiversity, various geographic regions, and Indigenous agropastoralist populations, e.g., the remote living tribe called Wiwa.

The Wiwa live in simple houses, consisting out of palm roofs and mud walls and floors. Water is taken from a nearby river or unprotected cisterns, which animals also have access to. There is no sanitation in place, no electricity, and no roads to reach their villages. Their access to health care is sparse. The next hospital is about 6 h walking distance. The Wiwa live as typical agropastoralists. Their diet is based on small-scale self-cultivated beans, rice, corn, and yucca, and relies on uncultivated fruits and plants, as well as eggs. Only infrequently are meat or fish consumed by these Indigenous people. Taken together, their subsistence way of life has all the typical characteristics of agropastoralists.

Due to extremely poor social and ecological conditions, environmental factors, and their traditional way of life (e.g., the use of top-dressing in agriculture), these Indigenous populations are at high risk of suffering from intestinal infections transmitted by bacteria, protozoan parasites, and/or helminths species.

When asked about their medical history, the Wiwa claim gastrointestinal infections to be one of their main problems. Therefore, an epidemiological assessment of the prevalence of enteropathogenic bacteria, protozoa, and helminths was previously conducted, indicating high colonization, infection, and infestation rates [22]. More than one hundred Indigenous people living in two small villages, Tezhumake (Department Cesar) and Siminke (Department La Guajira), volunteered to provide stool samples as study participants, representing 80% and 50% of the overall populations in the respective villages. About a dozen samples were included as control, originating from Colombians living in Valledupar, a larger city in the Department Cesar.

The previous study by our group [22] documented the high burden of gastrointestinal pathogens in the tested Indigenous individuals. Over 93% of all stool samples contained at least one of the tested pathogens. Overall, 79% of all stool samples contained protozoa, 69% helminths, and 41% bacteria. *G. intestinalis* (48%), Necator/hookworm (27%), and enteroaggregative *E. coli* (EAEC, 68%) were found to be the most dominant pathogens [22].

As known from previous assessments [23,24,25], however, molecular proof of pathogens, in particular, is not necessarily associated with clinical disease manifestation in high-endemicity settings [26], making adaptation processes likely. The composition of the gut microbiome might be an influencing factor for adaptation; however, it could also be directly associated with the presence of eukaryotic and prokaryotic enteric pathogens [13]. Geographic variation in gut microbiome compositions is a well-described phenomenon [27], most interestingly with widely dispersed resistance genes even in highly diverse gut microbiomes of uncontacted Indigenous populations [28]. Therefore, apart from the detection of single or multiple intestinal pathogen species, it remains unknown if the overall gut microbiomes of Colombian Indigenous individuals differ from those of larger city populations with higher socio-economic standards and access to healthcare facilities. It is further unclear if the presence of pathogens shapes the gut microbiomes of pathogen-colonized and/or -infected individuals. It could also be envisioned that dietary and environmental factors mainly shape the gut microbiomes of Indigenous populations and, thereby, the specific individual gut microbiome composition could be a risk factor for a higher pathogen burden.

To approach these questions epidemiologically, an analysis of the specific gut microbiome of Wiwa was conducted and associated with both self-reported clinical features and detected pathogenic microorganisms, as reported recently [22]. The aim was to characterize specific features of the gut microbiome of an isolated Indigenous Colombian population in comparison with non-Indigenous individuals, and to identify potential associations with and differences between village locations, gender, and age, in addition to clinical features such as the reporting of gastrointestinal symptoms. Dissimilarities in the bacterial distribution and the pathogen specific functional profiles of the microbiomes were also the focus of this study.

## 2. Materials and Methods

### 2.1. Study Population

The study population has been described previously [22]. In short, stool samples were collected from Indigenous volunteers from the remote Indigenous villages Tezhumake (*n* = 81, estimated population of 200, Department César) and Siminke (*n* = 45, estimated population of 60, Department La Guajira) between July and November 2014. In addition, 11 stool samples were provided by the Laboratory of Christian Gram (*n* = 7), the Hospital Rosario Pumarejo de Lopéz (*n* = 2), and voluntary Colombians (*n* = 2, non-Indigenous people) living in Valledupar. Two samples came from a health care center in Valledupar, serving the needs of the Wiwa, and were provided by Wiwa from Tezhumake.

Complete anthropometric, clinical, and physical examinations were performed by a physician in 102 cases and incomplete datasets were provided in an additional 34 cases. Specific assessed data of patients comprised date of birth, age, gender, height (cm), weight (kg), BMI/z-scores, location, stool pathogens (number of different species and identification on genus or species level), and specific symptoms. Official tables by the Organización Mundial de Salud 2006–2007 for the Colombian population were applied to evaluate average size, weight, and growth. Corrected by gender, individuals aged 0–19 years were scored by weight–size, weight–age, and size–age tables; further, body mass index (BMI) was calculated in line with WHO recommendations.

### 2.2. Previously Existing Diagnostic Information about the Samples

As detailed previously [22], stool samples were microscopically assessed at the Bernhard-Nocht Institute, Hamburg, Germany. In addition, PCR-based pathogen screening was performed at the Department of Microbiology & Hospital Hygiene of the Bundeswehr Hospital Hamburg, i.e., the German National Reference Centre for Tropical Pathogens in Hamburg, Germany [22]. In short, the samples were subjected to standard nucleic acid extraction by applying the QiaAMP DNA Stool Mini Kit (Qiagen, Hilden, Germany) as described by the manufacturer with subsequent assessment applied in-house [29,30], or commercial real-time PCR assays (the RidaGene real-time PCR kits, “EAEC”, “EHEC-EPEC”, and “‘ETEC-EIEC”, R-Biopharm, Darmstadt, Germany) targeting diarrheagenic *Escherichia coli*, *Salmonella* spp., *Shigella* spp./enteroinvasive *Escherichia coli*, *Campylobacter jejuni*, *Yersinia* spp., *Entamoeba histolytica*, *Giardia duodenalis*, *Cyclospora cayetanensis*, *Cryptosporidium* spp., *Ancylostoma* spp., *Ascaris lumbricoides*, *Necator americanus*, *Strongyloides stercoralis*, *Schistosoma* spp., *Trichuris trichiura*, *Taenia saginata*, *Taenia solium*, *Enterobius vermicularis*, and *Hymenolepis nana*. Performance characteristics of the applied PCR assays have been detailed elsewhere [29,30,31].

### 2.3. Library Preparation and Next Generation Sequencing (NGS) Analysis

The DNA extraction from all stool samples was previously described [22]. Prior to 16S rRNA gene amplicon library preparation, DNA concentration was again determined using Qubit and Nanodrop protocols. The amplicon PCR was started with 5 ng/μL of template DNA in 10 mM Tris buffer at pH 8.5. The V3/V4 region primers were used for the amplification of the 16S rRNA encoding gene [32], resulting in roughly 450 bp fragments. All other library preparation steps were performed according to the Illumina “16S Metagenomic Sequencing Library Preparation protocol”.

Further steps including PCR clean-up, Index PCR, PCR clean-up 2, library quantification, normalization, and pooling followed the above-mentioned protocols. Bioanalyzer DNA 1000 chips (Agilent Technologies, Santa Clara, CA, USA) and Qubit kits (Thermo Fischer Scientific, Waltham, MA, USA) allowed quantity and quality control of the final individual stool sample libraries and the final library pool. Two libraries were prepared from all samples. Ten percent PhiX control was spiked into each final pool and 5 pM of the final libraries were initially loaded on two 50 cycle V2 chemistry kits. An optimal cluster density and an even-read distribution among all samples was verified after sequencing both pools. Finally, each pool was subjected to full length sequencing on a 600 cycle V3 chemistry kit using an Illumina MiSeq machine. During each run, roughly 600 (k/mm^2^) clusters were sequenced. A total of 95% of all clusters passed the filter specs and sequencing lead to a mean of 16 Mio reads per run, of which again 95% passed the quality control. Amplicon sequencing of the 135 samples from the three locations generated over 22.31 million reads (6.39 × 10^9^ bp total), with 165,313 on average per sample. These reads were assembled, producing more than 9.84 million amplicons for the whole dataset (average 72,940 amplicons per sample).

### 2.4. Bioinformation Assessment of the NGS Reads

16S amplicons were assembled from the Illumina sequencing runs using the pandaseq program (version 2.11) [33]. Operational taxonomic units (OTUs) were then identified from these amplicons with USEARCH (version 6.1.544) [34], called from within the QIIME 1.9.1 pipeline [35]. To this end, the similarity of the amplicons to fragments in the 16S rRNA Greengenes (version 13.8) [36] database was used. In all cases, a cutoff of 97% identity was applied. Afterwards, low confidence OTUs were excluded via the remove_low_confidence_otus.py script [37]. Furthermore, contaminant OTUs, such as those classified as chloroplasts, mitochondria, or nonbacterial, were removed from the data. The obtained dataset was then processed with *phyloseq* (version 1.22.3) [38], a bioconductor package used to evaluate the ordination, abundance, and composition of the bacterial communities in the samples, within the R environment (version 3.4.3). Statistical significance of these analyses was assessed through the permanova (adonis) and Kruskal–Wallis tests, as implemented in the *vegan* package (version 2.4.6). Moreover, statistically significant differences between the communities from the different sample locations at the genus level were analyzed with the *DESeq2* R package [39], by applying a Wald test and the Benjamin–Hochberg correction for the *p*-values (false discovery rate cutoff: FDR < 0.01). Moreover, to observe the genera shared between the different communities, we followed the protocol developed in the *microbiome* R package (version 1.9.96), in order to calculate the core microbiomes for each sample group [40,41]. Finally, a PICRUSt analysis was carried out to predict the relative abundance of functional genes and pathways in the microbial communities (version 2.11) [42]. The obtained OTU (operational taxonomic unit) tables were then normalized by 16S rRNA copy number, and functional genes were predicted from the Kyoto Encyclopedia of Genes and Genomes (KEGG) database [43]. Finally, the results from the PICRUSt analysis and KEGG predictions were processed with STAMP (version 2.1.3) [44].

### 2.5. Data Deposit

The raw sequencing files have been deposited at the European Nucleotide Archive (ENA, https://www.ebi.ac.uk/ena, accessed on 24 January 2023) under accession number PRJEB43871.

### 2.6. Ethical Clearance

The study was performed in line with the declaration of Helsinki. It was approved by the Ethics Committee of Valledupar, César, Colombia on 11 August 2014 (Acta No 0022013). Written informed consent was obtained from each participant or from the parent or legal guardian of a child before participation. All participants were informed about their results and received treatment, if appropriate.

## 3. Results

### 3.1. Brief Demographic Sample Background

Demographic details of the assessed populations have previously been reported and are freely accessible [22]. Briefly, there was a 1:1 female-to-male ratio without associations with symptoms or pathogen detections in the previous study. A mean age (±standard deviation (SD)) of 24.6 (±18.2) years was recorded for the study population. Although only 12% of the tested individuals reported symptoms such as diarrhea (10%) or diarrhea combined with abdominal pain (2%), 82% of collected stool samples were fluid, pappy, or had a mucous texture, and 4 samples with PCR-confirmed diagnosis of *Entamoeba histolytica* were even bloody. Detections of bacterial, helminthic, and protozoan pathogens ranged between 39% and 60% [22] without association of reported symptoms and pathogen detections, while multiple infections were frequent in symptomatic individuals. At least 1 pathogen was detected in 93% of the assessed individuals, and a maximum of 9 were detected in 1 assessed volunteer, who was a 7-year-old boy. This corresponded well to the findings of higher proportions of both protozoan infections as well as multiple infections in children.

### 3.2. General Features of the Microbiome Dataset

The assembled amplicons were assigned to OTUs upon comparison with data from the Greengenes database (version 13.8), and filtered to keep only the high confidence assignments. From these, 43.92% of the total assembled fragments were attributed to Greengenes’ accessions with high-confidence assignments when using the open reference assignment method. Therefore, and to avoid spurious amplicons that might add bias to the results, low-quality samples (below 20,000 amplicons, with low read counts and a small number of assigned amplicons) were identified and excluded from further analyses. All follow-up analyses were carried out with data from the closed reference assignments, to maintain an agreement between the community-related results and those from the pathway analyses. The most predominant phyla in the individual samples that remained for further analyses were the *Proteobacteria*, with an average relative abundance of 23.85% (range from 0 to 87.89%), *Firmicutes* (range 6.46–70.36%; average 40.45%), and *Bacteroidetes* (average: 28.09%; range 0 to 55.88%). The whole microbiome of the selected samples also entailed 112 genera. Ten out of these 112 genera are shared by 80% of the subjects for a given group at the minimum detection threshold of 0.1% relative abundance, and therefore constitute the core microbiome: *Bacteroides*, *Prevotella*, *Clostridium*, *Coprococcus*, *Faecalibacterium*, *Oscillospira*, *Ruminococcus*, *Phascolarctobacterium*, *Succinivibrio*, and *Treponema* [41]. Specifically, the top five most abundant genera in the remaining samples were *Prevotella* (average between samples: 15.41%; range from 0.0 to 34.42%), *Faecalibacterium* (average 9.32%, range 0.0–46.07%), *Succinivibrio* (average 9.17%, range 0.0–35.16%), *Bacteroides* (average 3.31%, range 0.0 to 50.09%), and *Treponema* (average 2.92%, range 0.0 to 14.61%).

### 3.3. Composition of the Bacterial Communities by Location

**Figure 1:**
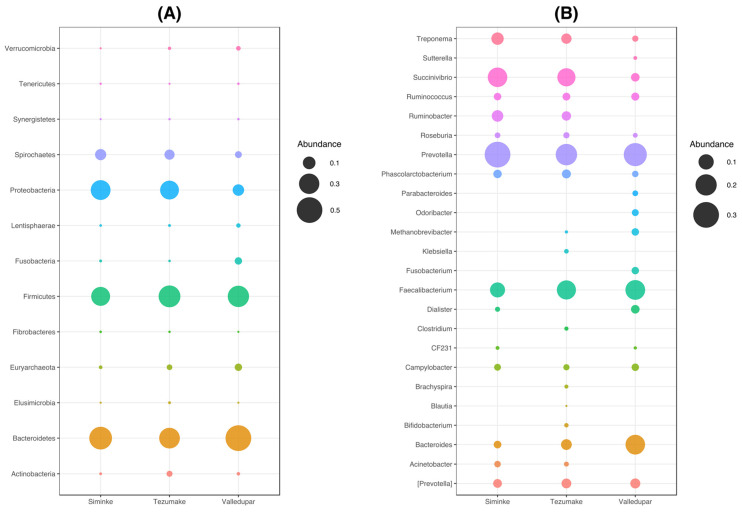
Community composition by location. (**A**) Phylum distribution, unfiltered. (**B**) Genera distribution (Genera > 2%).

First, we analyzed the relative abundance at the phylum level for the whole group. *Bacteroidetes*, *Firmicutes*, *Proteobacteria*, and *Spirochaetes* were among the most commonly represented phyla in all locations (Figure 1A). As shown in Figure 1A and Table A1, analysis of the Indigenous locations revealed that the top five most abundant phyla for the Siminke population were the *Bacteroidetes* (38.42%), *Proteobacteria* (28.25%), *Firmicutes* (25.51%), *Spirochaetes* (7.44%), and *Euryarchaeota* (0.22%). The most common phyla for the Indigenous population from Tezhumake were *Firmicutes* (35.04%), *Bacteroidetes* (31.18%), *Proteobacteria* (25.20%), *Spirochaetes* (5.84%), and *Actinobacteria* (1.33%; see Table A1 and Figure 1A). This result already highlights differential abundances, particularly of the *Firmicutes*, between Indigenous villages with the same agropastoralist subsistence. The urban population of Valledupar had the same top five most prominent phyla as Siminke, although with different relative abundances: *Bacteroidetes* (50.48%), *Firmicutes* (33.31%), *Proteobacteria* (7.92%), *Fusobacteria* (2.51%), and *Euryarchaeota* (2.52%; see Table 1 and Figure 1A). Of note, the urban microbiomes are remarkably dominated by *Bacteriodetes* (50.48%), whereas overall the abundance of *Proteobacteria* is 3–4 times higher in the Indigenous samples in relation to the urban village.

Furthermore, when considering the F/B ratio (Table A1), the urban samples are identical to the Siminke samples (0.66 in both populations). In the Tezhumake population the F/B ratio is twice as high compared to Siminke and Valledupar.

Second, we analyzed the relative abundance in all microbial communities at the genus level (Figure 1B, genera with relative abundance > 2%; Table A2, genera with relative abundance above 1%). The five major genera found in the Siminke population were *Prevotella* (30.09%), *Succinivibrio* (16.80%), *Faecalibacterium* (9.70%), *Treponema* (6.54%), and *Ruminobacter* (5.68%; Figure 1B, Table A2). Similarly, the most abundant genera in the Indigenous Tezhumake group were *Prevotella* (20.43%), *Faecalibacterium* (16.06%), *Succinivibrio* (14.40%), *Bacteroides* (4.93%), and *Treponema* (4.54%; Figure 1B, Table A2). The top five most abundant genera in the non-Indigenous Valledupar population were *Prevotella* (23.93%), *Faecalibacterium* (17.21%), *Bacteroides* (16.82%), and *Succinivibrio* (3.22%), and the genus *Dialister* (3.25%; Figure 1B, Table A2). Notice that in this case we are not considering the contested OTUs counted also as *Prevotella* (featured as “[Prevotella]” in Figure 1B; 4.29%). Together, this genus level comparison mainly revealed significant differences between the relative abundances of the top five ranking genera present in the Indigenous populations versus the Valledupar samples, apart from the absence of *Treponema* and presence of *Dialister* among the top five ranking genera of the Valledupar (Table A2, Kruskal–Wallis *p*-value 0.01 and 0.0336 respectively).

Extending the overview to all genera with relative abundance above 1% (Table A2), it is apparent that *Dialister* and CF231 (a genus within the *Paraprevotellaceae* family recently associated with fatty liver and higher BMI [45,46]) are missing in this category in the Tezhumake population stool samples, whereas *Klebsiella*, *Bifidobacteria*, *Clostridia*, *Brachyspira*, and *Blautia* can be found exclusively in the Tezhumake sample collection when considering relative abundance above 1%. *Ruminobacter* and *Acinetobacter* are not found among the genera with relative abundance above 1% in Valledupar individuals, whereas *Fusobacteria*, *Odoribacteria*, *Parabacterioides*, and *Sutterella* can only be found in the urban stool sample collection with the 1% cut-off used for this particular analysis. Consistent with these results, none of the exclusively found genera from the Tezhumake and Valledupar stool samples could be identified in the Siminke samples in the category with relative abundance above 1%.

**Table 1:** Differentially abundant taxa from the comparison between the different population groups. Comparison specifies the pair analyzed: Valledupar vs. Indigenous (VI), Valledupar vs. Tezhumake (VT), Valledupar vs. Siminke (VS), and Tezhumake vs. Siminke (TS). LFC indicates the Log2-fold changes between both sample groups, and the adjusted *p*-value was calculated with the Benjamin–Hochberg method to control the False Discovery Rate (FDR < 0.1), as implemented in the DESeq2 R library [39]. The OTU identifiers correspond to those pertaining to the Greengenes database, version 13.8 [47].

Comparison	Identifier	Phylum	Genus	LFC	*p*-Adjusted
VI	403,701	Firmicutes	*Dialister*	25.12	6.98 × 10^−4^
VI	4,454,586	Bacteroidetes	*Odoribacter*	5.52	8.42 × 10^−3^
VI	314,915	Bacteroidetes	*Parabacteroides*	−3.87	8.42 × 10^−3^
VI	562,244	Proteobacteria	*Sutterella*	−4.15	6.98 × 10^−4^
VT	403,701	Firmicutes	*Dialister*	24.59	3.34 × 10^−5^
VT	4,454,586	Bacteroidetes	*Odoribacter*	5.51	6.53 × 10^−4^
VT	562,244	Proteobacteria	*Sutterella*	−4.43	1.47 × 10^−4^
VS	314,915	Bacteroidetes	*Parabacteroides*	−3.83	5.99 × 10^−3^
VS	567,226	Bacteroidetes	*Prevotella*	−4.01	8.24 × 10^−3^
VS	531,614	Bacteroidetes	*Prevotella*	−4.05	2.38 × 10^−4^
VS	335,827	Firmicutes	*Butyrivibrio*	−4.16	1.13 × 10^−3^
VS	324,283	Bacteroidetes	*Prevotella*	−5.50	3.80 × 10^−4^
TS	173,726	Proteobacteria	*Sutterella*	2.89	3.56 × 10^−3^
TS	179,291	Firmicutes	*Faecalibacterium*	2.09	7.54 × 10^−3^
TS	353,173	Firmicutes	*Clostridium*	1.99	3.56 × 10^−3^
TS	324,283	Bacteroidetes	*Prevotella*	−3.14	8.17 × 10^−4^

Overall, the location-specific associations were calculated with a 75.76% accuracy employing a Random Forest Classifier (RFC) model.

**Figure 2:**
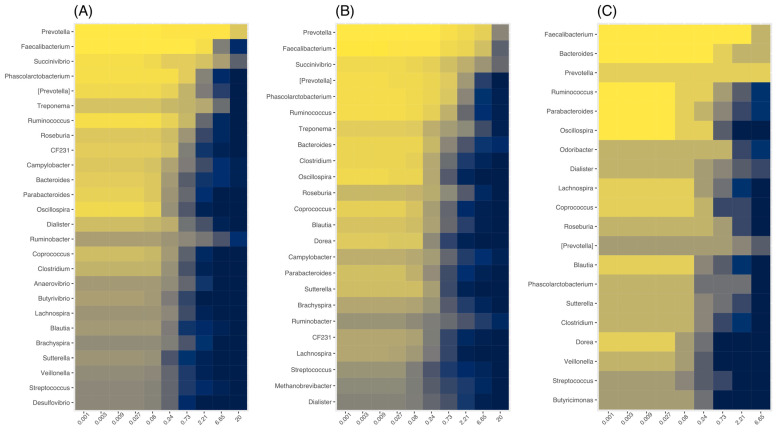
Core microbiomes by location: (**A**) Siminke; (**B**) Tezumake; (**C**) Valledupar. The x-axis indicates the detection threshold in relative abundances.

Next, the core microbiome (basically the number of taxa shared by a minimum number of individuals within one group) was analyzed. At the genus level, the Indigenous samples entailed the following nine genera: *Prevotella*, *Faecalibacterium*, *Succinivibrio*, *Phascolarctobacterium*, *Ruminococcus*, *Treponema*, *Bacteroides*, *Oscillospira*, and *Clostridium*. The core microbiome from the Siminke samples contained twelve genera without counting contested taxa (Figure 2A): *Prevotella*, *Faecalibacterium*, *Succinivibrio*, *Phascolarctobacterium*, *Treponema*, *Ruminococcus*, *Roseburia*, CF231, *Campylobacter*, *Bacteroides*, *Parabacteroides*, and *Oscillospira*. Similarly, the core microbiome in the Tezhumake Indigenous population consisted of ten genera (Figure 2B): *Prevotella*, *Faecalibacterium*, *Succinivibrio*, *Phascolarctobacterium*, *Ruminococcus*, *Treponema*, *Bacteroides*, *Clostridium*, *Oscillospira*, and *Coprococcus*. In contrast, the core microbiome in the urban population (Valledupar, Figure 2C) entailed the genera *Faecalibacterium*, *Bacteroides*, *Prevotella*, *Ruminococcus*, *Parabacteroides*, *Oscillospira*, *Lachnospira*, *Coprococcus*, and *Blautia*. Three genera were found distinctively in the core microbiomes of the Indigenous populations: *Phascolarctobacterium*, *Succinivibrio*, and *Treponema*, while *Blautia* and *Lachnospira* were predominant in the core microbiome of the urban individuals.

Furthermore, to extend our knowledge of the significant differences between these populations at the genus level, we carried out differential abundance tests over the OTU data. Upon applying a false discovery rate correction (FDR < 0.1), significant differences were found for all four pairs of comparisons (Table 1). Four genera were identified as significantly different between the urban (Valledupar) versus the Indigenous groups, three for the Valledupar and Tezhumake pair, five between Valledupar and Siminke, and finally four for Tezhumake versus Siminke (Table 1). In the first comparison, we observed that the genera *Dialister* and *Odoribacter* are significantly more abundant in the urban stool microbiomes from Valledupar with respect to the Indigenous samples. However, the genera *Parabacteroides* and *Suterella* where significantly less abundant in the Valledupar stool samples. Similarly, the genera *Dialister* and *Odoribacter* are more commonly found in the Valledupar samples (24.59 and 5.51 LFC, respectively), and *Suterella* is significantly more abundant in the Tezhumake Indigenous people. Differential comparisons between samples from Valledupar and Siminke revealed *Parabacteroides*, *Butyrivibrio*, and three OTUs belonging to the *Prevotella* genus were all significantly less abundant in the Valledupar stool microbiomes compared to those of the Siminke population. In addition, *Suterella*, *Faecalibacterium*, and *Clostridium* were significantly more abundant in the Tezhumake group, whereas the *Prevotella* genus was found to be less abundant when compared to the Siminke stool microbiome samples (Table 1).

### 3.4. Microbial Gut Diversity of the Indigenous and Urban Populations

**Figure A1:**
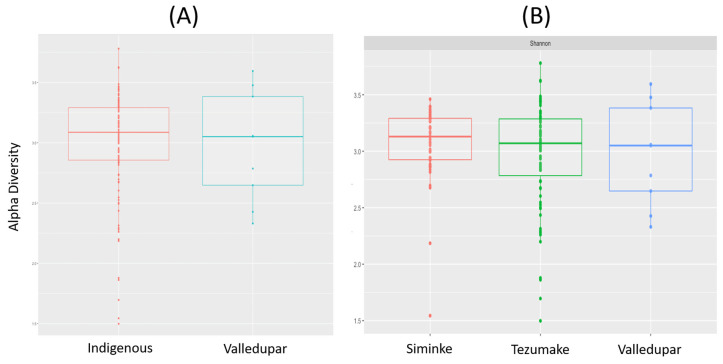
Shannon alpha diversities. (**A**): Indigenous vs. Valledupar; (**B**): All populations.

Next, we calculated and compared the Shannon diversity to identify possible significant differences in phylogenetic alpha diversities (within sample variation) between these different population groups. We found the non-Indigenous group (Valledupar) has less alpha diversity compared to the Indigenous individuals, indicating a rather uniform microbiome composition among all individual Valledupar samples (Figure A1A). The mean Shannon diversity was also lower when comparing Valledupar against the two individual Indigenous groups separately (Siminke and Tezhumake), while the overall diversity was larger in the Tezhumake versus the Valledupar. Conversely, the overall diversity was larger in the Valledupar than in the Siminke individuals (Figure A1B). Of note, the Kruskal–Wallis tests deemed these differences not significant in both cases (*p*-value < 0.7623 and *p*-value < 0.6399 for Figure A1A and B, respectively).

**Figure 3:**
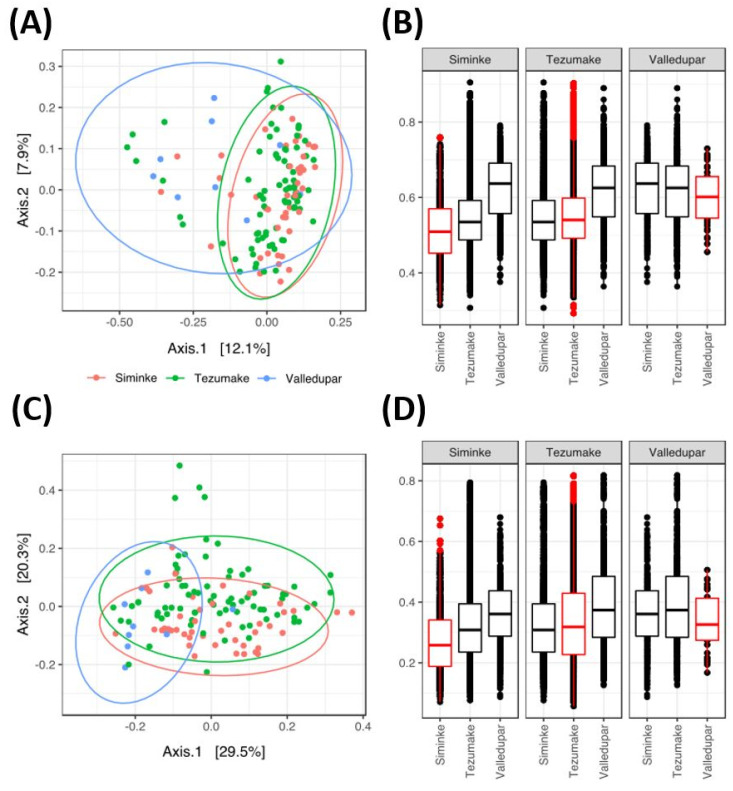
The Siminke and Tezumake microbiomes relative to that of the Valledupar population: (**A**,**C**) principal component analyses; (**B**,**D**) UniFrac distances among groups; (**A**,**B**) unweighted UniFrac distances; (**C**,**D**) weighted UniFrac.

Therefore, we extended our analyses to determine the community ß-diversity with unweighted UniFrac distances (qualitative approach based on presence/absence of taxa). Results shown in Figure 3A,B revealed the microbiome of the Indigenous population was significantly distant from that of the Valledupar group (permanova *p*-value < 1 × 10^−4^). These observations were confirmed when considering taxa abundances using weighted UniFrac metrics (based on presence/absence of taxa and relative abundance of taxa, permanova *p*-value < 5 × 10^−4^). In fact, the Siminke and Tezhumake microbiomes are similarly distant to those from Valledupar (unweighted UniFrac, permanova *p*-value 5 × 10^−4^; Figure 3A,B). However, when taking into account taxa abundances, the Tezhumake samples were more similar to those from the non-Indigenous Valledupar population than to the Siminke samples (weighted UniFrac, permanova *p*-value 5 × 10^−4^; Figure 3C,D).

### 3.5. Functional Composition of the Microbial Communities by Location

**Figure A2:**
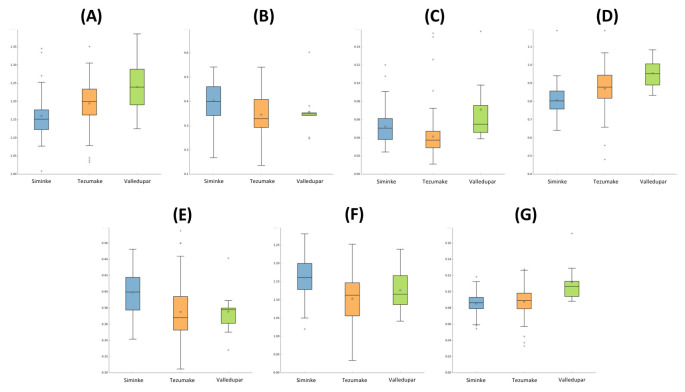
Significant pathways. (**A**) Arginine and proline metabolism, (**B**) lipopolysaccharide biosynthesis, (**C**) glycosphingolipid biosynthesis, (**D**) porphyrin and chlorophyll metabolism, (**E**) glycosyltransferases, (**F**) chaperones and folding catalysts, (**G**) bacterial toxins.

As a next step, we performed a PICRUSt analysis [42] to identify genera with significant differences between the three locations at the functional level. This allows inferring potential genetic capabilities and specific contributions of bacterial taxa to the imputed metagenome of all samples. Multiple group comparisons were carried out from the OTU genera data and their corresponding pathway-encoding genes for each genus. Employing a DESeq2 test with Benjamin–Hochberg FDR correction, we found five differentially abundant KEGG metabolic pathways (Table A3): Arginine and proline metabolism (KEGG id KO00330; adjusted *p*-value < 0.0266), Lipopolysaccharide biosynthesis (KO00540; adjusted *p*-value < 0.0379), Glycosphingolipid biosynthesis-ganglio series (KO00604; adjusted *p*-value < 0.0247), Porphyrin and chlorophyll metabolism (KO00860; adjusted *p*-value < 0.0102), and Glycosyltransferases (KO01003; adjusted *p*-value < 0.0297). Two significant pathways related to information processes were also identified: KO02042 Bacterial toxins and KO03110 Chaperones and folding catalysts. Genes related to the arginine and proline metabolism, as well as those from glycosphingolipid biosynthesis, porphyrin, and chlorophyll metabolism and bacterial toxins, were all enriched in the Valledupar samples (Figure A2A, C, D, and G, respectively). Those from the lipopolysaccharide biosynthesis, glycosyltransferases, and chaperones and folding catalysts were predominantly found in the Siminke population (Figure A2B, E, and F, respectively). The four significant pathways that were more represented in the Valledupar samples were linked to 143, 56, 52, and 38 unique genera for the glycosphingolipid biosynthesis, bacterial toxins, arginine and proline metabolism, and porphyrin and chlorophyll metabolism, respectively. Common to all these pathways were the following ten genera: *Bacillus*, *Citrobacter*, *Comamonas*, *Edwardsiella*, *Erwinia*, *Klebsiella*, *Paenibacillus*, *Pseudomonas*, *Serratia*, and *Trabulsiella*. In a similar manner, in the enriched pathways in the Siminke samples we identified 145, 134, and 12 unique genera for the chaperones and folding catalysts, lipopolysaccharide biosynthesis, and glycosyltransferases pathways, respectively. Eleven genera were common to these three pathways: *Achromobacter*, *Alloscardovia*, *Bacillus*, *Comamonas*, *Giesbergeria*, *Hydrogenophaga*, *Lysinibacillus*, *Paracoccus*, *Pigmentiphaga*, *Pseudomonas*, and *Sphingomonas*, while Elusimicrobium was exclusive to the lipopolysaccharide biosynthesis pathway, and another eleven genera were unique to the chaperones pathway (*Alkaliphilus*, *Butyrivibrio*, *Candidatus*, *Arthromitus*, *Fibrobacter*, *Finegoldia*, *Methanobrevibacter*, *Mogibacterium*, *Moryella*, *Proteiniclasticum*, and *Synergistes*). Next, when comparing the genera present in the significant pathways from Siminke against the genera from the pathways more abundant in the Valledupar group, we encountered 142 genera in common, four unique to the Siminke enriched pathways (*Actinobacillus*, *Methanobrevibacter*, *Methanosphaera*, and the Archaea genus vadinCA11), and a single genus exclusive to the Valledupar enriched pathways (*Peptococcus*). Finally, we filtered these genera for the corresponding sample groups (instead of via enriched pathways), and found that *Methanobrevibacter*, *Actinobacillus*, and vadinCA11 can be all found in individual samples of both Siminke and Valledupar, although *Methanobrevibacter* and *Actinobacillus* are present in more samples in the Siminke group (20 versus 4 samples for *Methanobrevibacter*; 3 versus 1 for *Actinobacillus*), while vadinCA11 can be identified in one sample for both groups, and *Methanosphaera* and *Peptococcus* appear only in Tezhumake individuals.

### 3.6. Microbial Communities in the Different Gender Groups

**Figure 4:**
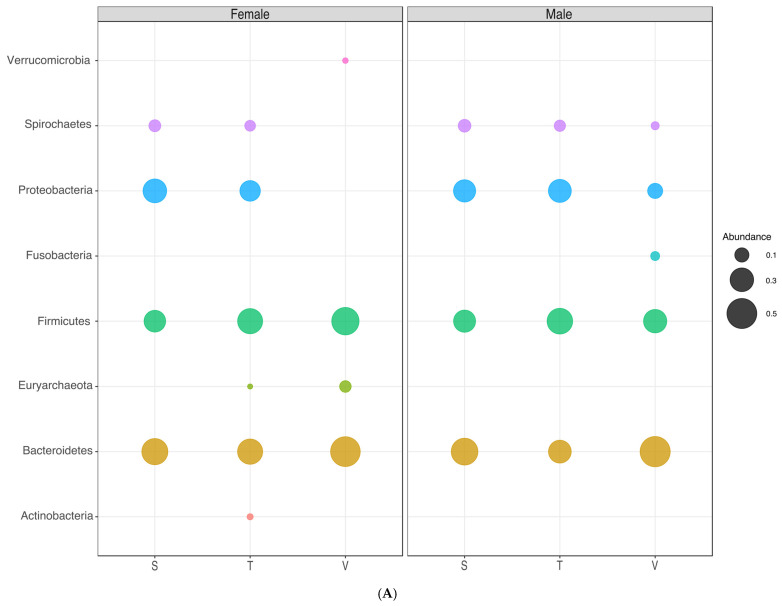

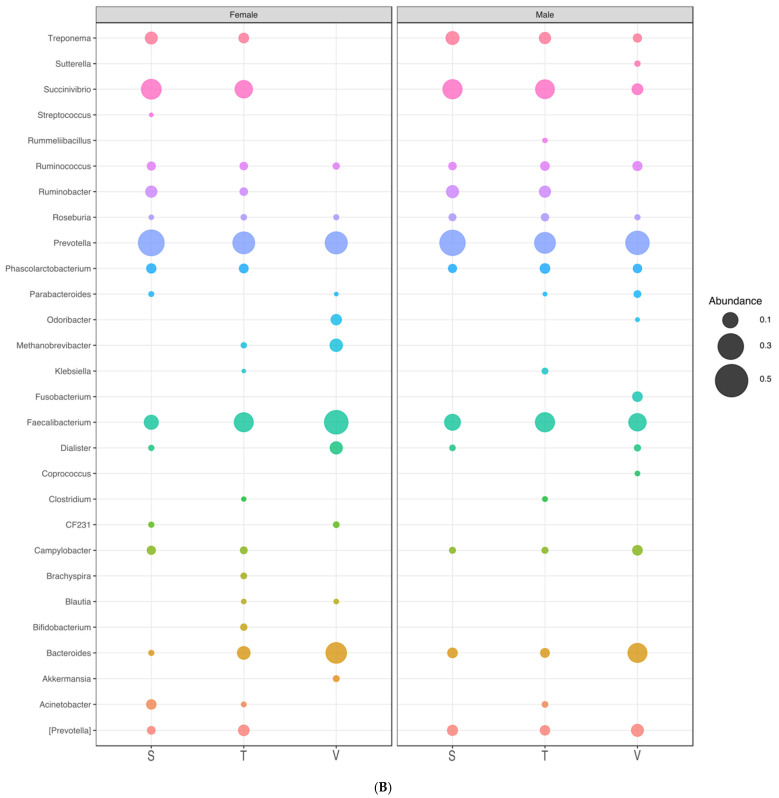
(**A**) Community composition by gender. Phylum distribution, unfiltered. Siminke (S), Tezhumake (T) and Valledupar (V). (**B**) Community composition by gender. Genera distribution (Genera > 2%). Siminke (S), Tezhumake (T) and Valledupar (V).

As a next step we analyzed the dataset for gender-specific differences in the microbiomes. The top four phyla in the female Indigenous samples were *Bacteriodetes*, *Proteobacteria*, *Firmicutes*, and *Spirochetes* (Figure 4A, Table A4). *Actinobacteria* and *Euryarcheota* have a higher abundance in females from Tezhumake compared to Siminke. The most abundant phyla from the female population of urban Valledupar are *Bacteriodetes*, *Firmicutes*, *Euryarcheota*, and *Verucomicrobiota*. The latter is found only below 1% abundance in Indigenous females, whereas *Proteobacteria*, *Spirochaetes*, and *Actinobacteria* are below 1% abundance in female urban stool microbiomes (Figure 4A, Table A4). No obvious differences among females from Siminke and Tezhumake were noted.

Male microbiomes from Tezhumake have an abundance of *Actinobacteria* and *Euryarcheota* below 1% compared with the females from the same location. No such differences were found among males and females from Siminke (Figure 4A, Table A4). Males from the urban site Valledupar differ in terms of higher abundance of *Proteobacteria*, *Spirochaetes*, and *Fusobacteria*, as well as lower abundances of *Euryarcheota* from female subjects.

From these results we calculated the *Firmicutes–Bacteroidetes* (FB) ratio for each gender in each population group, and found that the average FB ratios for females were 0.7061, 4.2882, and 0.9591 for the Siminke, Tezhumake, and Valledupar individuals, respectively, while in males these values were 0.7339, 1.3882, and 0.6299, respectively, for these same population groups. Kruskal–Wallis tests carried out from this data deemed differences in FB ratios between the three population groups as significant between genders (*p*-values: 0.0131 for the FB ratios in females; 1.3971 × 10^−5^ in males).

At the genera level, no difference in the abundance of the 10 most prevalent genera for the females in the Indigenous populations was noted (Figure 4B, Table A5). However, *Dialister*, *CF231*, *Bacteroides*, *Parabacteroides*, and *Streptococcus* are genera with an abundance above 1% in Siminke females compared to Tezhumake, where these genera did not appear in this category. Similarly, *Bifidobacterium*, *Brachyspira*, *Methanobrevibacter*, *Blautia*, *Clostridium*, and *Klebsiella* are more dominant in Tezhumake females compared to the Siminke female population (Figure 4B, Table A5). *Odoribacter* and *Akkermansia* are the only two genera which have an abundance above 1% exclusively in the urban female samples from Valledupar, whereas *Succinivibrio*, *Treponema*, *Ruminobacter*, *Acinetobacter*, *Phascolarctobacterium*, *Campylobacter*, *Streptococcus*, *Bifidobacterium*, *Brachyspira*, *Clostridium*, and *Klebsiella* are not found with an abundance above 1% in this urban female population. Genera abundance in males from all three locations is more diverse compared to the female microbiomes. Male Siminke microbiomes contain 12 genera with an abundance above 1%, whereas Tezhumake and Valledupar male microbiomes comprise 16 genera in that category (Figure 4B, Table A5). *Ruminobacter*, *Acinetobacter*, *Clostridium*, *Klebsiella*, and *Rummeliibacillus* are not found in Valledupar male samples in the abundance category above 1%. Similarly, *Dialister*, *Odoribacter*, *Coprococcus*, *Fusobacterium*, and *Sutterella* are not found in Tezhumake male samples, whereas *Acinetobacter*, *Parabacteroides*, *Clostridium*, *Klebsiella*, *Odoribacter*, *Coprococcus*, *Fusobacterium*, *Rummeliibacillus*, and *Suterella* are not found with an abundance above 1% in Siminke male microbiomes (Figure 4B, Table A5). Of note, Clostridium and *Klebsiella* are exclusively found above 1% abundance in male and female Tezhumake samples, whereas *Odoribacter* is exclusively categorized in the above 1% abundance category in female and male microbiomes from Valledupar. Some differences were noted by comparing females and males from the same location. In Siminke, *Acinetbacter*, *CF231*, *Parabacteroides*, and *Streptococcus* are found in female samples with an abundance above 1% if compared to the male microbiomes. In Tezhumake, *Bifidobacterium*, *Brachyspira*, *Methanobrevibacter*, and *Blautia* are more abundant in female microbiomes compared to male samples. More differences were noted among the samples from Valledupar, where the female microbiomes have a higher abundance of *CF231*, *Methanobrevibacter*, *Blautia*, and *Akkermansia* compared to male individuals. Males from Valledupar have a higher abundance in the category above 1% of *Succinivibrio*, *Treponema*, *Phascolarctobacterium*, *Campylobacter*, *Coprococcus*, *Fusobacterium*, and *Sutterella* (Figure 4B, Table A5).

### 3.7. Microbial Communities Associated with Different Age Groups

**Figure 5:**
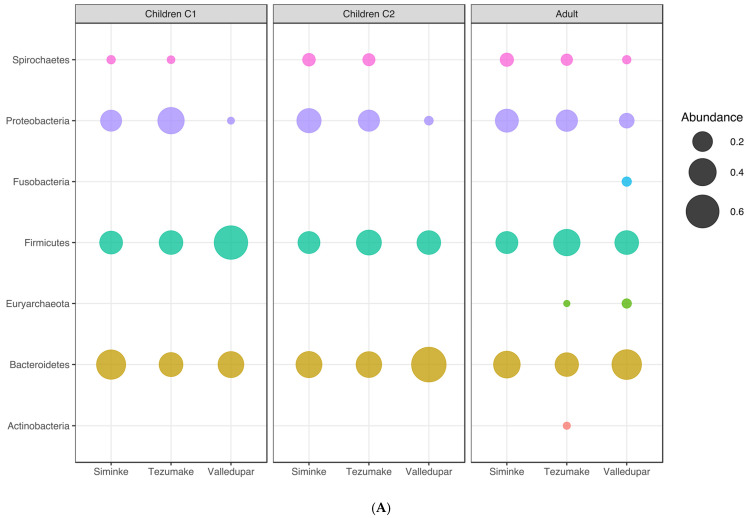

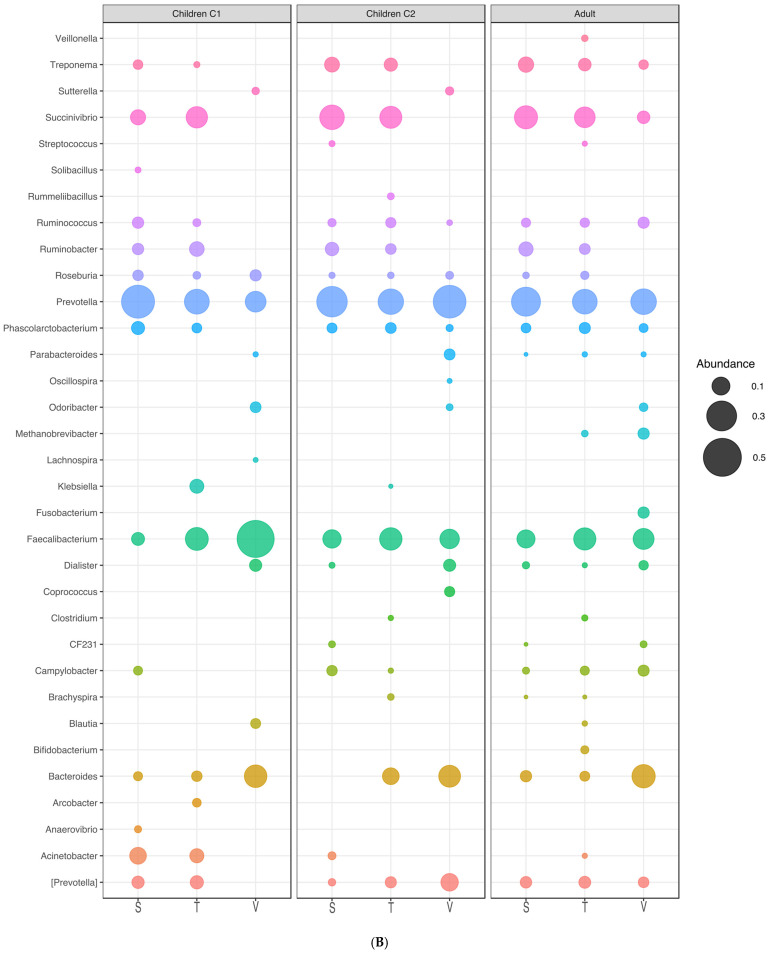
(**A**) Community composition by age group. Phylum distribution, unfiltered. Siminke (S), Tezhumake (T) and Valledupar (V). (**B**) Community composition by age group. B: Genera distribution (Genera > 2%). Siminke (S), Tezhumake (T) and Valledupar (V).

For simplicity, age groups were classified into children under 5 years of age (C1 age group), children between 5 and 17 years of age (C2 age group), and adults 18 years and older (C3 age group). When comparing the Indigenous populations versus Valledupar, we found that *Proteobacteria*, *Bacteroidetes*, and *Firmicutes* were the prevailing phyla in both groups (Table A6, Figure 5A). *Bacteroidetes* were present in larger proportions in the C2 and C3 age groups from the urban population of Valledupar, while the Firmicutes were most prevalent in the C1 age group for the same population (Table A6, Figure 5A). Further, we noticed the percentage of *Firmicutes* increased with age in the Indigenous groups, while it decreased from C1 to C2, and then remained more or less stable in the urban samples. The mean FB ratios increased with age in the Indigenous samples and decreased considerably from the C1 to C2 and C3 children and adults in Valledupar. *Spirochaetes* were proportionally more dominant in all Indigenous groups. Of note, no *Spirochaetes* were found in the urban children when filtering low abundant genera (below 1%). *Actinobacteria* were exclusively found in the adult C3 group from Indigenous locations, whereas *Fusobacteria* were exclusively found in the urban microbiome from Valledupar, if abundances lower than 1% were filtered out (Table A6, Figure 5A).

At the genera level, *Prevotella*, *Faecalibacterium*, and Bacteroides were found in all age groups in both Indigenous and urban populations (Table A7, Figure 5B). C1 children from Valledupar revealed a remarkably higher proportion of *Faecalibacterium*, which is equal in all other age groups from both locations. The proportion of *Bacteroides* is constantly larger in all age groups from the urban Valledupar microbiomes compared to the Indigenous samples. Ruminobacter was exclusively found in all Indigenous age groups within the above 1% abundance category, while *Campylobacter* is exclusive for the C1 and C3 Indigenous age groups and only appears in the microbiomes of both locations in the adult age group C3. For *Dialister* it was the opposite, as it is exclusive for the C1 and C2 age groups of the urban site and appears in both sites among the adult C3 microbiomes. *Bifidobacterium*, *Veillonella*, *Acinetobacter*, *Acrobacter*, and *Klebsiella* are exclusive in the Indigenous and C3 and C1 groups, respectively. Brachyspira and *Clostridium* were identified only in the Indigenous C2 and C3 age groups if the above 1% abundance level is considered. Two genera, *Odoribacter* and *Suterella*, are present only in urban samples in the above 1% abundance category. Further genera found exclusively in urban samples were *Lachnospira* (only observed in C1 children), *Coprococcus* and *Oscillospira* solely in C2 children, and *Fusobacterium* and CF23 in adults.

When analyzing the data individually for the two Indigenous populations, Siminke and Tezhumake displayed different abundances of *Bacteroidetes, Proteobacteria*, and *Firmicutes*, with *Bacteroidetes* always the most prevalent group in all ages for Siminke, and different phyla being the most common in the different age groups for Tezhumake: *Proteobacteria* in C1 children, *Bacteroidetes* in C2 children, and *Firmicutes* in C3 adults (Table A8, Figure 5A). Consequently, the mean FB ratio is almost twice as high in Tezhumake C1 children compared to C1 children from Siminke, while increasing from C1 to C2 children and then slightly increasing into adulthood for both Siminke and Valledupar. The differences in FB ratios between C2 children and C3 adults from the three populations are significant, with Kruskal–Wallis *p*-values of 0.0328 and 1.0214 × 10^−5,^ respectively.

As for genera exclusively present in Indigenous groups, *Streptococcus* appears in Siminke C2 children and Tezhumake C3 adults; *Klebsiella* in both children groups for Tezhumake; *Clostridium* in Tezhumake C2 children and C3 adults; *Brachyspira* also in Tezhumake C2 children and C3 adults, and also in Siminke adults; *Acinetobacter* in Siminke C1 and C2 children, and in Tezhumake C1 children and C3 adults; and, finally, *Ruminobacter* appears in all ages for both Indigenous groups (Table A9, Figure 5B).

There were significant differences when comparing the age-associated relative abundances of genera for the three populations (data not filtered for low abundant genera): Kruskal–Wallis *p*-values 3 × 10^−4^ for C1 children, 1 × 10^−4^ for the C2 children, and 0.0166 for C3 adults.

Overall, the age-specific associations were calculated with a 50% accuracy employing a Random Forest Classifier (RFC) model.

Furthermore, we refrained from a comparative analysis of microbial communities from the different locations in association with BMI, as our study did not provide enough individual samples to statistically and evenly cover all possible BMI categories (underweight, normal, pre-obesity, obesity, and overweight). Details are provided in Table A10.

### 3.8. Association between Specific Genera and Demographic and Clinical Metadata

Finally, and as data about co-occurring infections were available [22], we combined the microbiome OTU genera data with the infection data (symptoms, presence of pathogens, and parasites) to identify associations between dysbiosis and co-occurring infections. We found that changes in relative abundances of genera for the three populations were related to ETEC-positive (Kruskal–Wallis *p*-value < 9 × 10^−4^), EAEC-negative (*p*-value < 0.0058), and *Giardia*-positive (*p*-value < 0) individuals. Later, we combined the microbiome OTU genera data with all demographic (gender, age, location) and clinical data (BMI, symptoms, presence of pathogens and parasites) to assess the association of the microbiome data with these factors through generalized mixed models [48]. For simplicity, in these tests we utilized only the differentially abundant genera: *Sutterella*, *Faecalibacterium*, *Clostridium*, *Prevotella*, *Parabacteroides*, *Butyrivibrio*, *Dialister*, and *Odoribacter*, and the demographic and clinical data as response variables. As a result, we found that *Sutterella* was associated with individuals who tested positive for EHEC (*p*-value < 4.31 × 10^−2^) and EPEC (*p*-value < 4.64 × 10^−2^). *Faecalibacterium* was linked to Salmonellosis (*p*-value < 9.28 × 10^−4^), EPEC (*p*-value < 1.34 × 10^−2^) and Hymenolepis (*p*-value < 2.61 × 10^−2^). *Parabacteroides*, *Prevotella*, and *Butyrivibrio* were significant in those individuals with Enterobius, EPEC, and Salmonellosis, respectively (Table 2). In addition, we found that *Butyrivibrio*, *Sutterella*, *Dialister*, and *Faecalibacterium* were enriched in both Tezhumake and Valledupar populations, whereas *Clostridium* and *Prevotella* were Tezhumake-specific. *Odoribacter* and *Parabacteroides* were significantly present in the Valledupar individuals (Table 2). Finally, we identified genera that were enriched in the whole cohort for specific demographics, i.e., *Dialister* for those with symptoms and *Clostridium* in children under 5 years old (Table 2).

Overall, specific associations with the symptoms, EHEC, EPEC, *Salmonella*, *Enterobius*, and *Hymenolepsis* were calculated with 66.7, 81, 56.8, 95.5, 97.7, and 87.9% accuracy, respectively, employing a Random Forest Classifier (RFC) model.

## 4. Discussion

Several studies investigated age, gender, and nutritional associations with gut microbiomes of non-Western, urban populations from larger Colombian cities [49]. However, the Indigenous tribe called Wiwa, a restricted living and agropastoralist population in Colombia who lives in small villages under extremely poor nutritional, health, and hygiene conditions, have not been studied to date. The microbiomes of these Indigenous people potentially contain important bacterial species that have been lost during domestication and urbanization. However, this particular population in Colombia suffers from gastrointestinal infections that are rather common and widespread among these people. Consequently, in this research we investigated for the first time the stool microbiomes of the Wiwa population in two separate villages (Tezhumake and Siminke) to study the bacterial and functional diversity of their gut microbiomes. Furthermore, possible associations of morphometric data, epidemiological data, and gastrointestinal diseases with microbiome composition, always compared to a local urban population (Valledupar), were investigated.

The investigation provided a number of insights. First, locational-, age-, and gender-specific differences in the Firmicutes/Bacteriodetes ratio, core microbiome, and overall genera-level microbiome composition were shown, confirming previous reports on lifestyle-dependent enteric microbiomes in populations with a traditional lifestyle [10,12,13,14,15,17,18,19,20,27,28,50,51,52] or individuals exposed to nutrition associated with such a lifestyle [53]. Similar to the observations of earlier assessments of other Indigenous populations, alpha- and ß-diversity separated the urban site from the Indigenous locations in the assessment presented here. In part, poor significance was associated with the low number of samples taken from Colombians with an urban lifestyle. Urban microbiomes were dominated by *Bacteriodetes*, whereas Indigenous samples revealed a four times higher abundance of *Proteobacteria*. Even differences among the two compared Indigenous villages were noted, suggesting a considerable influence of minor peculiarities in community-specific lifestyle or prevalence of medical concomitant conditions, as suggested elsewhere [54].

When looking at the overall alpha-diversity (within sample variation), the urban individuals from Valledupar have a rather uniform microbiome composition across all individual samples. *Odoribacter* and *Parabacteroides* were exclusively identified in the microbiomes of the urban population of Valledupar. Individuals from both Indigenous Wiwa villages, in contrast, have a higher microbiome diversity. This is a consistent finding and emerging fact from many other global studies observing stool microbiomes in uncontacted Amerindian Yanomami [28], Papua New Guineans [55], and Tanzanian Hadza hunter–gatherers [56]. Such high diversity in Indigenous samples is clearly linked to traditional lifestyle, nutritional uptake, and lack of sanitation and personal hygiene [52]. In our collective, these alpha-diversity differences did not reach statistical significance, most likely due to uneven group sample sizes (126 Indigenous versus 9 urban). Thus, unweighted UniFrac distance analysis was conducted and showed with significance that the gut microbiome of both Wiwa villages was equally different from that of the urban samples. Including taxa abundance in a weighted UniFrac analysis confirmed these results, which also reached significance; however, the Tezhumake samples were more similar to those of Valledupar. These results matched well with observations from studying BaAka rainforest hunter–gatherers with their agriculturalist Bantu neighbors in the Central African Republic, which were compared to US American microbiome datasets from the Human Microbiome Project [19]. However, in our study, ß-diversity levels allowed a clear separation of urban from Indigenous populations.

Focusing on age and gender effects, it is noteworthy that *Clostridia* were exclusively found in children under the age of 5 years. This finding is in line with the previous observation that *Clostridioides difficile* is virtually absent in stool samples of Wiwa, irrespective of the abundance or absence of complaints of diarrhea [57,58,59].

One clear limitation of our and many other studies is the focus on pure taxonomy, as this does not immediately allow identification of causal members of the microbiota, and thereby does not fulfil the classical and molecular postulates of Koch [60]. However, even “omics” based microbiome studies, which at least allow a functional investigation of diverse microbiota, have a limitation as they rely on correctly and constantly curated gene annotation databases. PICRUSt (Phylogenetic Investigation of Communities by Reconstruction of Unobserved States) [42] is a computational method allowing inference of potential genetic functions and capabilities using our dataset. We found four pathways enriched in the urban microbiomes from Valledupar. Among them, Arginine-Proline metabolism was found to be enriched in microbiomes of animal-protein-rich food consumers (Western diet) [16], which fits with the urban lifestyle in Valledupar. *Peptococcus* (now renamed as *Peptostreptococcus*) was the single genus exclusive to the enriched pathways from Valledupar microbiomes. These bacteria are normal inhabitants of the gut; however, in humans with underlying disease they can turn into pathogenes causing infections. The genera *Actinobacillus*, *Methanobrevibacter*, and *Methanosphaera*, and the Archaea genus vadinCA11, were unique to the Siminke enriched pathways. Most likely these pathway-specific and location-predicted genera are associated with lifestyle and nutrition. Metagenomic shotgun sequencing would be a better method to identify such functional associations in microbiomes of diverse populations.

Focusing on associations of microbiome peculiarities and detections of enteric pathogens, a number of associations were observed with high predictive accuracy. In summary, *Sutterella* was associated with the abundance of enterohemorrhagic *Escherichia coli* (EHEC), *Faecalibacteria* was associated with enteropathogenic *Escherichia coli* (EPEC) and helminth species *Hymenolepsis nana* and *Enterobius vermicularis*. Finally, *Parabacteroides*, *Prevotella*, and *Butyrivibrio* were associated with salmonellosis, EPEC, and helminth infections. Of note, similar observations have been reported previously, both from epidemiological assessments and experimental veterinary medical assessments [61,62,63,64,65]. Such coincidences in the literature confirm the findings of the assessments presented here, although potential causal relations are still largely unknown. Other microbiome associations, e.g., those previously reported for *Entamoeba* infections [66], were, however, not observed in our study. Further, associations of the abundance of *Dialister* with gastrointestinal symptoms were recorded. The potential protective effects of Dialister against enteropathogens as observed in a veterinary challenge study might explain this observation [67].

The study has a number of limitations. First, the number of available samples was low, particularly regarding the Colombians with an urban lifestyle. This limitation weakened several significance levels. Second, limited available funding affected the sequence depth. Third, complaints of diarrhea are socially discouraged in the Wiwa community, so any associations with reported gastrointestinal disease have to be interpreted with care. In line with this, the study aim was focused on epidemiological associations with gastrointestinal disease of such high severity that it was considered as worth reporting by the Wiwa. Thereby, mild forms of gastroenteritis, which were considered as mere annoyances rather than real diseases by the Indigenous population, will most certainly have gone undetected by this approach. As, however, such mild disease variants are in turn a less severe threat to the Wiwa, and considering the nevertheless considerable proportion even of self-reported gastroenteritis cases, we feel confident that the chosen case definition meets the needs of the assessed study population quite well.

## 5. Conclusions

Dysbiotic alterations of the gut microbiome in the Indigenous population of the Colombian Wiwa with frequent episodes of self-reported gastrointestinal disorders were confirmed. Thereby, epidemiological and pathogen-specific associations were shown. The information provided by this explorative assessment suggests microbiome alterations associated with the clinical conditions of the Indigenous population of the Wiwa. Confirmatory studies are needed to assess potential causal relationships. Future applicability of artificial intelligence applications will most likely be helpful for the assessment of complex associations at the microbiome level. In particular, regarding the fact that the samples were collected 10 years ago, a follow-up assessment might provide further insights into the temporary stability of the local microbiome compositions, especially due to the fact that the living conditions of the Wiwa have not principally changed in the meantime. In the case of a stable situation, the results could be used as baseline values in order to monitor potential effects and side effects induced by preventive medical interventions against gastrointestinal infection risks to the microbiomes of the Wiwa. Apart from their general microbiome dysbiosis, all volunteers were treated according to their results. In addition, a mass deworming took place, which is supposed to be repeated on a regular basis. The implementation of water filters was processed and evaluated, but had only a small impact [57].

## Figures and Tables

**Table 2:** Genera significantly associated with demographic and clinical data. OTU genera abundances (log10-corrected) were combined with demographic and clinical data (“category”) and evaluated through generalized mixed linear models. Results are shown for genera under the significance cutoff (*p*-value < 0.05).

Genus	Category	*p*-Value
*Butyrivibrio*	BMI:Overweight	0.0401
*Butyrivibrio*	location:Tezhumake	0.0007
*Butyrivibrio*	location:Valledupar	0.0009
*Butyrivibrio*	Salmonella:Yes	0.0204
*Clostridium*	Age:Child Under 5	0.0244
*Clostridium*	location:Tezhumake	0.0011
*Dialister*	location:Tezhumake	0.0209
*Dialister*	location:Valledupar	0.0451
*Dialister*	symptoms.presence:Yes	0.0480
*Faecalibacterium*	EPEC:Yes	0.0134
*Faecalibacterium*	Hymenolepis:Yes	0.0261
*Faecalibacterium*	location:Tezhumake	0.0276
*Faecalibacterium*	location:Valledupar	0.0301
*Faecalibacterium*	Salmonella:Yes	0.0009
*Odoribacter*	BMI:Obesity	0.0494
*Odoribacter*	BMI:Overweight	0.0169
*Odoribacter*	location:Valledupar	1.26 × 10^−14^
*Parabacteroides*	Enterobius:Yes	0.0349
*Parabacteroides*	location:Valledupar	0.0093
*Prevotella*	EPEC:Yes	0.0134
*Prevotella*	location:Tezhumake	0.0392
*Sutterella*	EHEC:Yes	0.0431
*Sutterella*	EPEC:Yes	0.0464
*Sutterella*	location:Tezhumake	0.0020
*Sutterella*	location:Valledupar	0.0133

## Data Availability

All relevant data are provided within the manuscript and its appendices. Raw data can be made available on reasonable request. The raw sequencing files have been deposited at the European Nucleotide Archive (ENA, https://www.ebi.ac.uk/ena, accessed on 24 January 2023) under the accession number: PRJEB43871.

## Appendix A

**Table A1:** Phylum distribution in the three locations. Data indicate relative abundances (percentages of OTU counts). Phyla with relative abundances lower than 1% were filtered out.

Phylum	Siminke	Tezhumake	Valledupar
Bacteroidetes	38.42%	31.18%	50.48%
Proteobacteria	28.25%	25.20%	7.92%
Firmicutes	25.51%	35.04%	33.31%
Spirochaetes	7.44%	5.84%	2.06%
Euryarchaeota	0.22%	1.10%	2.52%
Fusobacteria	0.06%	0.02%	2.51%
Actinobacteria	0.05%	1.33%	0.17%
Lentisphaerae	0.03%	0.06%	0.46%
Fibrobacteres	0.02%	0.01%	0.00%
Tenericutes	0.01%	0.00%	0.01%
Verrucomicrobia	0.00%	0.15%	0.54%
Elusimicrobia	0.00%	0.06%	0.00%
Synergistetes	0.00%	0.01%	0.01%
F/B ratio	0.66	1.12	0.66

**Table A2:** Genera distribution in the three locations. Data indicate relative abundances (percentages of OTU counts). Genera with relative abundances lower than 1% were filtered out.

Genus	Siminke	Tezhumake	Valledupar
*Prevotella*	30.09%	20.43%	23.93%
*Succinivibrio*	16.80%	14.40%	3.22%
*Faecalibacterium*	9.70%	16.06%	17.21%
*Treponema*	6.54%	4.54%	1.88%
*Ruminobacter*	5.68%	3.78%	0.00%
[Prevotella]	3.35%	4.12%	4.29%
*Phascolarctobacterium*	3.12%	3.48%	1.99%
*Bacteroides*	2.64%	4.93%	16.82%
*Ruminococcus*	2.55%	2.73%	2.86%
*Campylobacter*	2.20%	1.87%	2.51%
*Acinetobacter*	2.03%	1.44%	0.00%
*Roseburia*	1.69%	1.89%	1.40%
*Dialister*	1.48%	0.00%	3.25%
*CF231*	1.19%	0.00%	1.11%
*Klebsiella*	0.00%	1.34%	0.00%
*Bifidobacterium*	0.00%	1.29%	0.00%
*Clostridium*	0.00%	1.25%	0.00%
*Brachyspira*	0.00%	1.21%	0.00%
*Methanobrevibacter*	0.00%	1.10%	2.51%
*Blautia*	0.00%	1.03%	0.00%
*Fusobacterium*	0.00%	0.00%	2.51%
*Odoribacter*	0.00%	0.00%	2.23%
*Parabacteroides*	0.00%	0.00%	1.70%
*Sutterella*	0.00%	0.00%	1.19%

**Table A3:** Metabolic and signaling pathways differentially represented in the three locations. *Identifier* indicates the unique KEGG id (Kanehisa et al., 2010 [43]), while *Level 1* and *Level 3* are the corresponding KEGG pathway categories, and Unique genera are OTUs pertaining to a given pathway.

Identifier	Level 1	Level 3	Corrected *p*-Values	Unique Genera
KO00330	Metabolism	Arginine and proline metabolism	0.0266	52
KO00540	Metabolism	Lipopolysaccharide biosynthesis	0.0379	134
KO00604	Metabolism	Glycosphingolipid biosynthesis—ganglio series	0.0247	143
KO00860	Metabolism	Porphyrin and chlorophyll metabolism	0.0102	38
KO01003	Metabolism	Glycosyltransferases	0.0297	12
KO02042	Environmental Information Processing	Bacterial toxins	0.0237	56
KO03110	Genetic Information Processing	Chaperones and folding catalysts	0.0017	145

**Table A4:** Phylum distribution for the gender groups in the three locations. Data indicate relative abundances (percentages of OTU counts). Phyla with relative abundances lower than 1% were filtered out.

Phylum	Female			Male		
	Siminke	Tezhumake	Valledupar	Siminke	Tezhumake	Valledupar
Bacteroidetes	37.39%	34.44%	49.32%	39.36%	27.82%	51.06%
Proteobacteria	30.33%	22.25%	0.00%	26.36%	28.23%	11.40%
Firmicutes	24.97%	34.23%	41.35%	26.00%	35.88%	29.29%
Spirochaetes	6.91%	5.54%	0.00%	7.92%	6.14%	3.08%
Actinobacteria	0.00%	1.87%	0.00%	0.00%	0.00%	0.00%
Euryarchaeota	0.00%	1.45%	6.46%	0.00%	0.00%	0.00%
Verrucomicrobia	0.00%	0.00%	1.63%	0.00%	0.00%	0.00%
Fusobacteria	0.00%	0.00%	0.00%	0.00%	0.00%	3.77%
F/B ratio	0.67	0.99	0.84	0.66	1.29	0.57

**Table A5:** Genera distribution for the gender groups for the three locations: Siminke (S), Tezhumake (T), and Valledupar (V). Data indicate relative abundances (percentages of OTU counts). Genera with relative abundances lower than 1% were filtered out.

Genus	Female			Male		
	S	T	V	S	T	V
*Prevotella*	30.68%	21.47%	21.97%	29.55%	19.36%	24.91%
*Succinivibrio*	17.39%	13.27%	0.00%	16.26%	15.56%	4.82%
*Faecalibacterium*	8.39%	15.94%	25.34%	10.90%	16.18%	13.15%
*Treponema*	5.93%	3.84%	0.00%	7.10%	5.26%	2.82%
*Ruminobacter*	5.19%	2.43%	0.00%	6.14%	5.17%	0.00%
*Acinetobacter*	3.63%	1.32%	0.00%	0.00%	1.57%	0.00%
*Phascolarctobacterium*	3.53%	3.17%	0.00%	2.75%	3.79%	2.95%
*Campylobacter*	2.76%	2.03%	0.00%	1.69%	1.71%	3.77%
*Ruminococcus*	2.74%	2.39%	1.80%	2.38%	3.07%	3.39%
[Prevotella]	2.47%	4.62%	0.00%	4.16%	3.61%	5.98%
*Dialister*	1.42%	0.00%	6.27%	1.53%	0.00%	1.73%
*CF231*	1.41%	0.00%	1.57%	0.00%	0.00%	0.00%
*Bacteroides*	1.39%	6.65%	18.80%	3.78%	3.17%	15.83%
*Parabacteroides*	1.33%	0.00%	1.08%	0.00%	1.10%	2.01%
*Roseburia*	1.24%	1.50%	1.38%	2.10%	2.29%	1.41%
*Streptococcus*	1.08%	0.00%	0.00%	0.00%	0.00%	0.00%
*Bifidobacterium*	0.00%	1.82%	0.00%	0.00%	0.00%	0.00%
*Brachyspira*	0.00%	1.59%	0.00%	0.00%	0.00%	0.00%
*Methanobrevibacter*	0.00%	1.45%	6.46%	0.00%	0.00%	0.00%
*Blautia*	0.00%	1.27%	1.26%	0.00%	0.00%	0.00%
*Clostridium*	0.00%	1.19%	0.00%	0.00%	1.31%	0.00%
*Klebsiella*	0.00%	1.06%	0.00%	0.00%	1.63%	0.00%
*Odoribacter*	0.00%	0.00%	4.46%	0.00%	0.00%	1.12%
*Akkermansia*	0.00%	0.00%	1.63%	0.00%	0.00%	0.00%
*Coprococcus*	0.00%	0.00%	0.00%	0.00%	0.00%	1.27%
*Fusobacterium*	0.00%	0.00%	0.00%	0.00%	0.00%	3.77%
*Rummeliibacillus*	0.00%	0.00%	0.00%	0.00%	1.23%	0.00%
*Sutterella*	0.00%	0.00%	0.00%	0.00%	0.00%	1.47%

**Table A6:** Phylum distribution for the age groups in the Indigenous and Valledupar locations. Data indicate relative abundances (percentages of OTU counts). Phyla with relative abundances lower than 1% were filtered out.

Phylum	Children (C1)		Children (C2)		Adult (C3)	
	Indigenous	Valledupar	Indigenous	Valledupar	Indigenous	Valledupar
Firmicutes	28.73%	62.21%	30.63%	29.58%	32.84%	29.74%
Bacteroidetes	35.74%	35.60%	35.31%	66.43%	32.43%	47.64%
Proteobacteria	32.28%	1.96%	25.78%	3.26%	25.37%	10.47%
Spirochaetes	2.63%	0.00%	6.99%	0.00%	6.84%	2.94%
Actinobacteria	0.00%	0.00%	0.00%	0.00%	1.30%	0.00%
Euryarchaeota	0.00%	0.00%	0.00%	0.00%	1.06%	3.78%
Fusobacteria	0.00%	0.00%	0.00%	0.00%	0.00%	3.77%
F/B ratio	0.80	1.72	0.87	0.45	1.01	0.62

**Table A7:** Genera distribution for the gender groups in the Indigenous and Valledupar locations. Data indicate relative abundances (percentages of OTU counts). Genera with relative abundances lower than 1% were filtered out.

Genus	Children (C1)		Children (C2)		Adult (C3)	
	Indigenous	Valledupar	Indigenous	Valledupar	Indigenous	Valledupar
*Prevotella*	26.17%	13.71%	24.45%	36.36%	23.07%	21.49%
*Succinivibrio*	11.84%	0.00%	16.84%	0.00%	15.01%	4.72%
*Faecalibacterium*	12.66%	47.52%	14.37%	11.95%	13.67%	13.92%
*Treponema*	1.88%	0.00%	5.71%	0.00%	5.68%	2.70%
*Ruminobacter*	5.56%	0.00%	4.01%	0.00%	4.51%	0.00%
[Prevotella]	4.94%	0.00%	3.06%	9.39%	4.10%	3.30%
*Phascolarctobacterium*	3.66%	0.00%	3.26%	1.69%	3.34%	2.42%
*Bacteroides*	2.94%	16.24%	5.98%	15.18%	3.23%	17.46%
*Ruminococcus*	2.64%	0.00%	2.79%	1.30%	2.60%	3.74%
*Campylobacter*	1.50%	0.00%	1.85%	0.00%	2.17%	3.65%
*Roseburia*	2.39%	3.74%	1.50%	1.96%	1.89%	0.00%
*Dialister*	0.00%	4.44%	0.00%	4.36%	1.40%	2.67%
*Bifidobacterium*	0.00%	0.00%	0.00%	0.00%	1.27%	0.00%
*Parabacteroides*	0.00%	1.21%	0.00%	3.45%	1.15%	1.20%
*Blautia*	0.00%	2.94%	0.00%	0.00%	1.13%	0.00%
*Veillonella*	0.00%	0.00%	0.00%	0.00%	1.12%	0.00%
*Clostridium*	0.00%	0.00%	1.00%	0.00%	1.07%	0.00%
*Methanobrevibacter*	0.00%	0.00%	0.00%	0.00%	1.05%	3.76%
*Acinetobacter*	6.87%	0.00%	0.00%	0.00%	1.05%	0.00%
*Brachyspira*	0.00%	0.00%	1.23%	0.00%	1.04%	0.00%
*Fusobacterium*	0.00%	0.00%	0.00%	0.00%	0.00%	3.77%
*Odoribacter*	0.00%	3.46%	0.00%	1.60%	0.00%	2.24%
*CF231*	0.00%	0.00%	0.00%	0.00%	0.00%	1.62%
*Arcobacter*	1.46%	0.00%	0.00%	0.00%	0.00%	0.00%
*Coprococcus*	0.00%	0.00%	0.00%	3.05%	0.00%	0.00%
*Klebsiella*	3.80%	0.00%	0.00%	0.00%	0.00%	0.00%
*Lachnospira*	0.00%	1.15%	0.00%	0.00%	0.00%	0.00%
*Oscillospira*	0.00%	0.00%	0.00%	1.16%	0.00%	0.00%
*Rummeliibacillus*	0.00%	0.00%	1.11%	0.00%	0.00%	0.00%
*Sutterella*	0.00%	1.79%	0.00%	2.10%	0.00%	0.00%

**Table A8:** Phylum distribution through the age groups for the three locations.

Phylum	Children (C1)			Children (C2)			Adult (C3)		
	Siminke	Tezhumake	Valledupar	Siminke	Tezhumake	Valledupar	Siminke	Tezhumake	Valledupar
Bacteroidetes	46.17%	29.95%	35.60%	36.09%	34.95%	66.43%	38.10%	28.93%	47.64%
Proteobacteria	22.91%	37.49%	1.96%	31.09%	23.32%	3.26%	27.86%	23.82%	10.47%
Firmicutes	27.32%	29.52%	62.21%	25.17%	33.16%	29.58%	25.33%	37.48%	29.74%
Spirochaetes	2.95%	2.45%	0.00%	7.39%	6.81%	0.00%	8.32%	5.91%	2.94%
Actinobacteria	0.00%	0.00%	0.00%	0.00%	0.00%	0.00%	0.00%	2.08%	0.00%
Euryarchaeota	0.00%	0.00%	0.00%	0.00%	0.00%	0.00%	0.00%	1.56%	3.78%
Fusobacteria	0.00%	0.00%	0.00%	0.00%	0.00%	0.00%	0.00%	0.00%	3.77%
F/B ratio	0.59	0.99	1.75	0.70	0.95	0.45	0.66	1.30	0.62

**Table A9:** Genera distribution through the age groups for the three locations: Siminke (S), Tezhumake (T), and Valledupar (V).

Genus	Children (C1)			Children (C2)			Adult (C3)		
	S	T	V	S	T	V	S	T	V
*Prevotella*	37.13%	20.09%	13.71%	31.36%	21.25%	36.36%	28.10%	19.96%	21.49%
*Succinivibrio*	6.95%	14.56%	0.00%	19.65%	15.53%	0.00%	17.26%	13.61%	4.72%
*Faecalibacterium*	5.02%	16.90%	47.52%	10.54%	16.15%	11.95%	10.19%	15.82%	13.92%
*Treponema*	2.74%	1.40%	0.00%	6.73%	5.24%	0.00%	7.17%	4.75%	2.70%
*Ruminobacter*	3.81%	6.53%	0.00%	5.45%	3.34%	0.00%	6.16%	3.49%	0.00%
[Prevotella]	4.49%	5.19%	0.00%	1.78%	3.65%	9.39%	3.92%	4.20%	3.30%
*Bacteroides*	2.45%	3.20%	16.24%	0.00%	8.53%	15.18%	3.75%	2.90%	17.46%
*Phascolarctobacterium*	5.10%	2.85%	0.00%	2.96%	3.40%	1.69%	2.82%	3.66%	2.42%
*Ruminococcus*	3.81%	1.99%	0.00%	2.11%	3.11%	1.30%	2.53%	2.63%	3.74%
*Dialister*	0.00%	0.00%	4.44%	1.37%	0.00%	4.36%	1.73%	1.20%	2.67%
*Campylobacter*	2.42%	0.00%	0.00%	3.20%	1.23%	0.00%	1.66%	2.48%	3.65%
*Roseburia*	3.27%	1.90%	3.74%	1.43%	1.53%	1.96%	1.52%	2.13%	0.00%
*Parabacteroides*	0.00%	0.00%	1.21%	0.00%	0.00%	3.45%	1.03%	1.23%	1.20%
*CF231*	0.00%	0.00%	0.00%	1.59%	0.00%	0.00%	1.03%	0.00%	1.62%
*Brachyspira*	0.00%	0.00%	0.00%	0.00%	1.54%	0.00%	1.03%	1.05%	0.00%
*Bifidobacterium*	0.00%	0.00%	0.00%	0.00%	0.00%	0.00%	0.00%	2.04%	0.00%
*Methanobrevibacter*	0.00%	0.00%	0.00%	0.00%	0.00%	0.00%	0.00%	1.55%	3.76%
*Veillonella*	0.00%	0.00%	0.00%	0.00%	0.00%	0.00%	0.00%	1.51%	0.00%
*Clostridium*	0.00%	0.00%	0.00%	0.00%	1.22%	0.00%	0.00%	1.42%	0.00%
*Blautia*	0.00%	0.00%	2.94%	0.00%	0.00%	0.00%	0.00%	1.24%	0.00%
*Acinetobacter*	8.53%	5.94%	0.00%	2.00%	0.00%	0.00%	0.00%	1.21%	0.00%
*Streptococcus*	0.00%	0.00%	0.00%	1.38%	0.00%	0.00%	0.00%	1.20%	0.00%
*Fusobacterium*	0.00%	0.00%	0.00%	0.00%	0.00%	0.00%	0.00%	0.00%	3.77%
*Odoribacter*	0.00%	0.00%	3.46%	0.00%	0.00%	1.60%	0.00%	0.00%	2.24%
*Anaerovibrio*	1.67%	0.00%	0.00%	0.00%	0.00%	0.00%	0.00%	0.00%	0.00%
*Arcobacter*	0.00%	2.27%	0.00%	0.00%	0.00%	0.00%	0.00%	0.00%	0.00%
*Coprococcus*	0.00%	0.00%	0.00%	0.00%	0.00%	3.05%	0.00%	0.00%	0.00%
*Klebsiella*	0.00%	5.88%	0.00%	0.00%	1.07%	0.00%	0.00%	0.00%	0.00%
*Lachnospira*	0.00%	0.00%	1.15%	0.00%	0.00%	0.00%	0.00%	0.00%	0.00%
*Oscillospira*	0.00%	0.00%	0.00%	0.00%	0.00%	1.16%	0.00%	0.00%	0.00%
*Rummeliibacillus*	0.00%	0.00%	0.00%	0.00%	1.60%	0.00%	0.00%	0.00%	0.00%
*Solibacillus*	1.35%	0.00%	0.00%	0.00%	0.00%	0.00%	0.00%	0.00%	0.00%
*Sutterella*	0.00%	0.00%	1.79%	0.00%	0.00%	2.10%	0.00%	0.00%	0.00%

**Table A10:** Phylum distribution (relative abundances) for the BMI and age groups for the three locations: Siminke (S), Tezhumake (T), and Valledupar (V). Valledupar data were only available for adults with normal BMI (18.5–24.9).

Phylum	Children (C1)				Children (C2)						Adult (C3)				
	Risk of Overweight		Overweight		Normal		Overweight		Obesity		Normal			Pre-Obesity	
	S	T	S	T	S	T	S	T	S	T	S	T	V	S	T
Bacteroidetes	50.49%	35.98%	46.97%	36.03%	38.28%	30.00%	31.71%	40.50%	25.30%	37.94%	39.71%	29.73%	50.15%	32.56%	35.20%
Proteobacteria	14.06%	29.11%	14.02%	25.19%	27.94%	25.95%	38.00%	21.92%	30.24%	24.90%	28.79%	20.68%	16.58%	23.63%	26.17%
Firmicutes	34.18%	32.50%	25.87%	35.29%	25.36%	34.52%	25.84%	28.16%	31.29%	33.48%	23.51%	40.48%	16.49%	33.61%	28.36%
Spirochaetes	0.00%	0.00%	12.28%	3.32%	8.16%	7.52%	4.10%	6.05%	13.02%	3.53%	7.53%	6.21%	4.52%	9.91%	9.06%
Euryarchaeota	0.00%	0.00%	0.00%	0.00%	0.00%	0.00%	0.00%	2.45%	0.00%	0.00%	0.00%	2.20%	0.00%	0.00%	1.11%
Fusobacteria	0.00%	0.00%	0.00%	0.00%	0.00%	0.00%	0.00%	0.00%	0.00%	0.00%	0.00%	0.00%	11.30%	0.00%	0.00%
Elusimicrobia	0.00%	1.41%	0.00%	0.00%	0.00%	0.00%	0.00%	0.00%	0.00%	0.00%	0.00%	0.00%	0.00%	0.00%	0.00%
F/B ratio	0.68	0.90	0.55	0.98	0.66	1.15	0.81	0.70	1.24	0.88	0.59	1.36	0.33	1.03	0.81

## References

[1] Reyes A., Semenkovich N.P., Whiteson K., Rohwer F., Gordon J.I. (2012). Going viral: Next-generation sequencing applied to phage populations in the human gut. Nat. Rev. Microbiol..

[2] Hamad I., Sokhna C., Raoult D., Bittar F. (2012). Molecular detection of eukaryotes in a single human stool sample from Senegal. PLoS ONE.

[3] Hoffmann C., Dollive S., Grunberg S., Chen J., Li H., Wu G.D., Lewis J.D., Bushman F.D. (2013). Archaea and fungi of the human gut microbiome: Correlations with diet and bacterial residents. PLoS ONE.

[4] Dusko Ehrlich S., MetaHIT Consortium (2010). Métagénomique du microbiote intestinal: Les applications potentielles [Metagenomics of the intestinal microbiota: Potential applications]. Gastroenterol. Clin. Biol..

[5] Kau A.L., Ahern P.P., Griffin N.W., Goodman A.L., Gordon J.I. (2011). Human nutrition, the gut microbiome and the immune system. Nature.

[6] Nicholson J.K., Holmes E., Kinross J., Burcelin R., Gibson G., Jia W., Pettersson S. (2012). Host-gut microbiota metabolic interactions. Science.

[7] Kamada N., Seo S.U., Chen G.Y., Núñez G. (2013). Role of the gut microbiota in immunity and inflammatory disease. Nat. Rev. Immunol..

[8] Sarkar A., Harty S., Lehto S.M., Moeller A.H., Dinan T.G., Dunbar R.I.M., Cryan J.F., Burnet P.W.J. (2018). The Microbiome in Psychology and Cognitive Neuroscience. Trends Cogn. Sci..

[9] Rampelli S., Turroni S., Mallol C., Hernandez C., Galván B., Sistiaga A., Biagi E., Astolfi A., Brigidi P., Benazzi S. (2021). Components of a Neanderthal gut microbiome recovered from fecal sediments from El Salt. Commun. Biol..

[10] Obregon-Tito A.J., Tito R.Y., Metcalf J., Sankaranarayanan K., Clemente J.C., Ursell L.K., Zech Xu Z., Van Treuren W., Knight R., Gaffney P.M. (2015). Subsistence strategies in traditional societies distinguish gut microbiomes. Nat. Commun..

[11] Gupta V.K., Paul S., Dutta C. (2017). Geography, Ethnicity or Subsistence-Specific Variations in Human Microbiome Composition and Diversity. Front. Microbiol..

[12] Hansen M.E.B., Rubel M.A., Bailey A.G., Ranciaro A., Thompson S.R., Campbell M.C., Beggs W., Dave J.R., Mokone G.G., Mpoloka S.W. (2019). Population structure of human gut bacteria in a diverse cohort from rural Tanzania and Botswana. Genome Biol..

[13] Rubel M.A., Abbas A., Taylor L.J., Connell A., Tanes C., Bittinger K., Ndze V.N., Fonsah J.Y., Ngwang E., Essiane A. (2020). Lifestyle and the presence of helminths is associated with gut microbiome composition in Cameroonians. Genome Biol..

[14] Dubois G., Girard C., Lapointe F.J., Shapiro B.J. (2017). The Inuit gut microbiome is dynamic over time and shaped by traditional foods. Microbiome.

[15] Girard C., Tromas N., Amyot M., Shapiro B.J. (2017). Gut Microbiome of the Canadian Arctic Inuit. mSphere.

[16] De Filippo C., Di Paola M., Ramazzotti M., Albanese D., Pieraccini G., Banci E., Miglietta F., Cavalieri D., Lionetti P. (2017). Diet, Environments, and Gut Microbiota. A Preliminary Investigation in Children Living in Rural and Urban Burkina Faso and Italy. Front. Microbiol..

[17] Afolayan A.O., Ayeni F.A., Moissl-Eichinger C., Gorkiewicz G., Halwachs B., Högenauer C. (2019). Impact of a Nomadic Pastoral Lifestyle on the Gut Microbiome in the Fulani Living in Nigeria. Front. Microbiol..

[18] Conteville L.C., Oliveira-Ferreira J., Vicente A.C.P. (2019). Gut Microbiome Biomarkers and Functional Diversity Within an Amazonian Semi-Nomadic Hunter-Gatherer Group. Front. Microbiol..

[19] Gomez A., Petrzelkova K.J., Burns M.B., Yeoman C.J., Amato K.R., Vlckova K., Modry D., Todd A., Jost Robinson C.A., Remis M.J. (2016). Gut Microbiome of Coexisting BaAka Pygmies and Bantu Reflects Gradients of Traditional Subsistence Patterns. Cell Rep..

[20] Sánchez-Quinto A., Cerqueda-García D., Falcón L.I., Gaona O., Martínez-Correa S., Nieto J., Santoyo I.G. (2020). Gut Microbiome in Children from Indigenous and Urban Communities in México: Different Subsistence Models, Different Microbiomes. Microorganisms.

[21] Escobar J.S., Klotz B., Valdes B.E., Agudelo G.M. (2014). The gut microbiota of Colombians differs from that of Americans, Europeans and Asians. BMC Microbiol..

[22] Kann S., Bruennert D., Hansen J., Mendoza G.A.C., Gonzalez J.J.C., Quintero C.L.A., Hanke M., Hagen R.M., Backhaus J., Frickmann H. (2020). High Prevalence of Intestinal Pathogens in Indigenous in Colombia. J. Clin. Med..

[23] Frickmann H., Schwarz N.G., Rakotozandrindrainy R., May J., Hagen R.M. (2015). PCR for enteric pathogens in high-prevalence settings. What does a positive signal tell us?. Infect. Dis..

[24] Krumkamp R., Sarpong N., Schwarz N.G., Adlkofer J., Loag W., Eibach D., Hagen R.M., Adu-Sarkodie Y., Tannich E., May J. (2015). Gastrointestinal infections and diarrheal disease in Ghanaian infants and children: An outpatient case-control study. PLoS Negl. Trop. Dis..

[25] Eibach D., Krumkamp R., Hahn A., Sarpong N., Adu-Sarkodie Y., Leva A., Käsmaier J., Panning M., May J., Tannich E. (2016). Application of a multiplex PCR assay for the detection of gastrointestinal pathogens in a rural African setting. BMC Infect. Dis..

[26] Zautner A.E., Groß U., Emele M.F., Hagen R.M., Frickmann H. (2017). More Pathogenicity or Just More Pathogens?-On the Interpretation Problem of Multiple Pathogen Detections with Diagnostic Multiplex Assays. Front. Microbiol..

[27] Yatsunenko T., Rey F.E., Manary M.J., Trehan I., Dominguez-Bello M.G., Contreras M., Magris M., Hidalgo G., Baldassano R.N., Anokhin A.P. (2012). Human gut microbiome viewed across age and geography. Nature.

[28] Clemente J.C., Pehrsson E.C., Blaser M.J., Sandhu K., Gao Z., Wang B., Magris M., Hidalgo G., Contreras M., Noya-Alarcón Ó. (2015). The microbiome of uncontacted Amerindians. Sci. Adv..

[29] Wiemer D., Loderstaedt U., von Wulffen H., Priesnitz S., Fischer M., Tannich E., Hagen R.M. (2011). Real-time multiplex PCR for simultaneous detection of *Campylobacter jejuni*, *Salmonella*, *Shigella* and *Yersinia* species in fecal samples. Int. J. Med. Microbiol..

[30] Köller T., Hahn A., Altangerel E., Verweij J.J., Landt O., Kann S., Dekker D., May J., Loderstädt U., Podbielski A. (2020). Comparison of commercial and in-house real-time PCR platforms for 15 parasites and microsporidia in human stool samples without a gold standard. Acta Trop..

[31] Hahn A., Luetgehetmann M., Landt O., Schwarz N.G., Frickmann H. (2017). Comparison of one commercial and two in-house TaqMan multiplex real-time PCR assays for detection of enteropathogenic, enterotoxigenic and enteroaggregative *Escherichia coli*. Trop. Med. Int. Health.

[32] Herlemann D.P., Labrenz M., Jürgens K., Bertilsson S., Waniek J.J., Andersson A.F. (2011). Transitions in bacterial communities along the 2000 km salinity gradient of the Baltic Sea. ISME J..

[33] Masella A.P., Bartram A.K., Truszkowski J.M., Brown D.G., Neufeld J.D. (2012). PANDAseq: Paired-end assembler for illumina sequences. BMC Bioinform..

[34] Edgar R.C. (2010). Search and clustering orders of magnitude faster than BLAST. Bioinformatics.

[35] Caporaso J.G., Kuczynski J., Stombaugh J., Bittinger K., Bushman F.D., Costello E.K., Fierer N., Peña A.G., Goodrich J.K., Gordon J.I. (2010). QIIME allows analysis of high-throughput community sequencing data. Nat. Methods.

[36] McDonald D., Price M.N., Goodrich J., Nawrocki E.P., DeSantis T.Z., Probst A., Andersen G.L., Knight R., Hugenholtz P. (2012). An improved Greengenes taxonomy with explicit ranks for ecological and evolutionary analyses of bacteria and archaea. ISME J..

[37] Comeau A.M., Douglas G.M., Langille M.G. (2017). Microbiome Helper: A Custom and Streamlined Workflow for Microbiome Research. mSystems.

[38] McMurdie P.J., Holmes S. (2013). Phyloseq: An R package for reproducible interactive analysis and graphics of microbiome census data. PLoS ONE.

[39] Love M.I., Huber W., Anders S. (2014). Moderated estimation of fold change and dispersion for RNA-seq data with DESeq2. Genome Biol..

[40] Salonen A., Salojärvi J., Lahti L., de Vos W.M. (2012). The adult intestinal core microbiota is determined by analysis depth and health status. Clin. Microbiol. Infect..

[41] Shetty S.A., Hugenholtz F., Lahti L., Smidt H., de Vos W.M. (2017). Intestinal microbiome landscaping: Insight in community assemblage and implications for microbial modulation strategies. FEMS Microbiol. Rev..

[42] Langille M.G., Zaneveld J., Caporaso J.G., McDonald D., Knights D., Reyes J.A., Clemente J.C., Burkepile D.E., Vega Thurber R.L., Knight R. (2013). Predictive functional profiling of microbial communities using 16S rRNA marker gene sequences. Nat. Biotechnol..

[43] Kanehisa M., Goto S., Furumichi M., Tanabe M., Hirakawa M. (2010). KEGG for representation and analysis of molecular networks involving diseases and drugs. Nucleic Acids Res..

[44] Parks D.H., Tyson G.W., Hugenholtz P., Beiko R.G. (2014). STAMP: Statistical analysis of taxonomic and functional profiles. Bioinformatics.

[45] Yun Y., Kim H.N., Kim S.E., Heo S.G., Chang Y., Ryu S., Shin H., Kim H.L. (2017). Comparative analysis of gut microbiota associated with body mass index in a large Korean cohort. BMC Microbiol..

[46] Stanislawski M.A., Lozupone C.A., Wagner B.D., Eggesbø M., Sontag M.K., Nusbacher N.M., Martinez M., Dabelea D. (2018). Gut microbiota in adolescents and the association with fatty liver: The EPOCH study. Pediatr. Res..

[47] DeSantis T.Z., Hugenholtz P., Larsen N., Rojas M., Brodie E.L., Keller K., Huber T., Dalevi D., Hu P., Andersen G.L. (2006). Greengenes, a chimera-checked 16S rRNA gene database and workbench compatible with ARB. Appl. Environ. Microbiol..

[48] Paniagua Voirol L.R., Weinhold A., Johnston P.R., Fatouros N.E., Hilker M. (2020). Legacy of a Butterfly’s Parental Microbiome in Offspring Performance. Appl. Environ. Microbiol..

[49] García-Vega Á.S., Corrales-Agudelo V., Reyes A., Escobar J.S. (2020). Diet Quality, Food Groups and Nutrients Associated with the Gut Microbiota in a Nonwestern Population. Nutrients.

[50] Sharma A.K., Petrzelkova K., Pafco B., Jost Robinson C.A., Fuh T., Wilson B.A., Stumpf R.M., Torralba M.G., Blekhman R., White B. (2020). Traditional Human Populations and Nonhuman Primates Show Parallel Gut Microbiome Adaptations to Analogous Ecological Conditions. mSystems.

[51] Jha A.R., Davenport E.R., Gautam Y., Bhandari D., Tandukar S., Ng K.M., Fragiadakis G.K., Holmes S., Gautam G.P., Leach J. (2018). Gut microbiome transition across a lifestyle gradient in Himalaya. PLoS Biol..

[52] Horwood P.F., Tarantola A., Goarant C., Matsui M., Klement E., Umezaki M., Navarro S., Greenhill A.R. (2019). Health Challenges of the Pacific Region: Insights From History, Geography, Social Determinants, Genetics, and the Microbiome. Front. Immunol..

[53] Ruggles K.V., Wang J., Volkova A., Contreras M., Noya-Alarcon O., Lander O., Caballero H., Dominguez-Bello M.G. (2018). Changes in the Gut Microbiota of Urban Subjects during an Immersion in the Traditional Diet and Lifestyle of a Rainforest Village. mSphere.

[54] Afolayan A.O., Adebusoye L.A., Cadmus E.O., Ayeni F.A. (2020). Insights into the gut microbiota of Nigerian elderly with type 2 diabetes and non-diabetic elderly persons. Heliyon.

[55] Martínez I., Stegen J.C., Maldonado-Gómez M.X., Eren A.M., Siba P.M., Greenhill A.R., Walter J. (2015). The gut microbiota of rural papua new guineans: Composition, diversity patterns, and ecological processes. Cell. Rep..

[56] Smits S.A., Leach J., Sonnenburg E.D., Gonzalez C.G., Lichtman J.S., Reid G., Knight R., Manjurano A., Changalucha J., Elias J.E. (2017). Seasonal cycling in the gut microbiome of the Hadza hunter-gatherers of Tanzania. Science.

[57] Kann S., Concha G., Hartmann M., Köller T., Alker J., Schotte U., Kreienbrock L., Frickmann H., Warnke P. (2022). Only Low Effects of Water Filters on the Enteric Carriage of Gastrointestinal Pathogen DNA in Colombian Indigenous People. Microorganisms.

[58] Weinreich F., Hahn A., Eberhardt K.A., Kann S., Köller T., Warnke P., Dupke S., Dekker D., May J., Frickmann H. (2022). Multicentric Evaluation of SeeGene Allplex Real-Time PCR Assays Targeting 28 Bacterial, Microsporidal and Parasitic Nucleic Acid Sequences in Human Stool Samples. Diagnostics.

[59] Kann S., Concha G., Köller T., Alker J., Schotte U., Hahn A., Frickmann H., Warnke P. (2022). Enteric Bacteria and Parasites with Pathogenic Potential in Individuals of the Colombian Indigenous Tribe Kogui. Microorganisms.

[60] Rosen C.E., Palm N.W. (2017). Functional Classification of the Gut Microbiota: The Key to Cracking the Microbiota Composition Code: Functional classifications of the gut microbiota reveal previously hidden contributions of indigenous gut bacteria to human health and disease. Bioessays.

[61] Mir R.A., Schaut R.G., Allen H.K., Looft T., Loving C.L., Kudva I.T., Sharma V.K. (2019). Cattle intestinal microbiota shifts following *Escherichia coli* O157:H7 vaccination and colonization. PLoS ONE.

[62] Wang X., Wu X., Cong X., Ren J., Li J., Zhu J., Dai M., Hrabchenko N., Du Y., Qi J. (2022). The functional role of fecal microbiota transplantation on *Salmonella* Enteritidis infection in chicks. Vet. Microbiol..

[63] Lin D., Song Q., Liu J., Chen F., Zhang Y., Wu Z., Sun X., Wu X. (2022). Potential Gut Microbiota Features for Non-Invasive Detection of Schistosomiasis. Front. Immunol..

[64] Paz E.A., Chua E.G., Hassan S.U., Greeff J.C., Palmer D.G., Liu S., Lamichhane B., Sepúlveda N., Liu J., Tay C.Y. (2022). Bacterial communities in the gastrointestinal tract segments of helminth-resistant and helminth-susceptible sheep. Anim. Microbiome.

[65] Latorre J.D., Adhikari B., Park S.H., Teague K.D., Graham L.E., Mahaffey B.D., Baxter M.F.A., Hernandez-Velasco X., Kwon Y.M., Ricke S.C. (2018). Evaluation of the Epithelial Barrier Function and Ileal Microbiome in an Established Necrotic Enteritis Challenge Model in Broiler Chickens. Front. Vet. Sci..

[66] Morton E.R., Lynch J., Froment A., Lafosse S., Heyer E., Przeworski M., Blekhman R., Ségurel L. (2015). Variation in Rural African Gut Microbiota Is Strongly Correlated with Colonization by *Entamoeba* and Subsistence. PLoS Genet..

[67] López-Colom P., Castillejos L., Rodríguez-Sorrento A., Puyalto M., Mallo J.J., Martín-Orúe S.M. (2019). Efficacy of medium-chain fatty acid salts distilled from coconut oil against two enteric pathogen challenges in weanling piglets. J. Anim. Sci. Biotechnol..

